# Dynamics of promoter bivalency and RNAP II pausing in mouse stem and differentiated cells

**DOI:** 10.1186/s12861-018-0163-7

**Published:** 2018-02-20

**Authors:** Anna Mantsoki, Guillaume Devailly, Anagha Joshi

**Affiliations:** 1 Division of Developmental Biology, The Roslin Institute and Royal (Dick) School of Veterinary Studies, University of Edinburgh, Easter Bush Campus, Midlothian, EH25 9RG UK; 2 GenPhySE, Université de Toulouse, INRA, INPT, ENVT, Toulouse, Haute-Garonne France

**Keywords:** ES cells, Chromatin, Bivalent, RNA pol II, Pausing, Gene expression

## Abstract

**Background:**

Mammalian embryonic stem cells display a unique epigenetic and transcriptional state to facilitate pluripotency by maintaining lineage-specification genes in a poised state. Two epigenetic and transcription processes involved in maintaining poised state are bivalent chromatin, characterized by the simultaneous presence of activating and repressive histone methylation marks, and RNA polymerase II (RNAPII) promoter proximal pausing. However, the dynamics of histone modifications and RNAPII at promoters in diverse cellular contexts remains underexplored.

**Results:**

We collected genome wide data for bivalent chromatin marks H3K4me3 and H3K27me3, and RNAPII (8WG16) occupancy together with expression profiling in eight different cell types, including ESCs, in mouse. The epigenetic and transcription profiles at promoters grouped in over thirty clusters with distinct functional identities and transcription control.

**Conclusion:**

The clustering analysis identified distinct bivalent clusters where genes in one cluster retained bivalency across cell types while in the other were mostly cell type specific, but neither showed a high RNAPII pausing. We noted that RNAPII pausing is more associated with active genes than bivalent genes in a cell type, and was globally reduced in differentiated cell types compared to multipotent.

**Electronic supplementary material:**

The online version of this article (10.1186/s12861-018-0163-7) contains supplementary material, which is available to authorized users.

## Background

Mammalian embryonic stem cells (ESCs) can differentiate to cell types with distinct functionalities in response to external and internal cues through epigenetic and transcription control [1, 2]. Genome-wide profiling of epigenetic features, chromatin accessibility, transcription factor (TF) DNA binding and gene expression across hundreds of cell types and tissues formed the building blocks for systematic studies of the regulatory control during development and differentiation [3–6], where specific combinations of histone marks reflect the expression status [7]. Multivariate statistical models [8–10] have proven highly effective in analysing combinatorial patterns of multiple epigenetic datasets in individual [11] or multiple cell types [12] and tissues [13] to identify distinct chromatin states. One particularly interesting chromatin state is the poised or bivalent state [14, 15] exhibiting both activating (H3K4me3) and silencing (H3K27me3) histone marks. This chromatin state was enriched at the promoters of many developmental genes in ESCs and to a lesser extent in differentiated cell types [15–20]. Bivalent promoters in ESCs are thought to maintain genes in a poised state safeguarding them from terminal silencing and retaining their plasticity to activate or silence during differentiation [21, 22].

Another mechanism associated with maintaining an intermediate transcription status is promoter-proximal accumulation of transcriptionally engaged but paused RNA polymerase II (RNAPII) [23, 24]. The RNAPII promoter proximal pausing was initially thought to be a property of developmental genes [9, 25–27]. Paused RNAPII and bivalent genes indeed showed a high overlap [28], though bivalent promoters show divergent RNAPII pausing signatures [23]. In order to systematically study the interplay of bivalent chromatin and RNAPII pausing associated with gene expression across cell types, we integrated histone modification data (H3K4me3, H3K27me3), RNAPII (8WG16) binding and expression profiling at the promoter regions of genes in eight different cell types, including ESCs, in mouse.

## Results

### Hierarchical clustering H3K27me3, H3K4me3, RNAPII and gene expression data in eight cell types

**Fig. 1:**
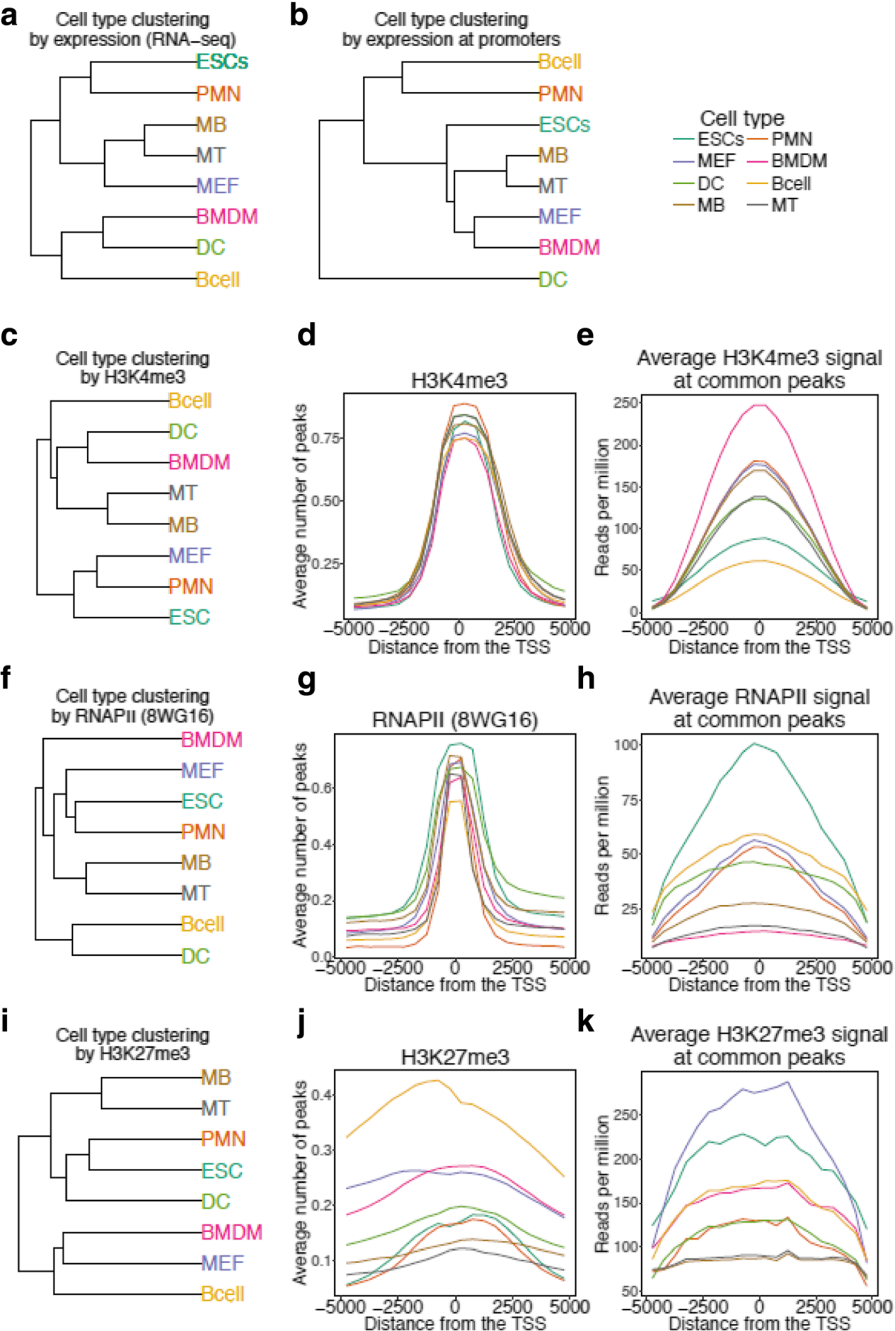
Expression, H3K4me3, H3K27me3 and RNAPII (8WG16) signatures at promoters of 22,179 genes in eight mouse cell types. **a** Hierarchical clustering of normalised expression values (see Methods) across eight cell types results in a tree where biological relationships between cell types are largely reconstituted. **b** Hierarchical clustering of average normalized RNA-seq signal (reads per million -RPM) across the gene promoters (±5 kb) for eight cell types. **c** Hierarchical clustering of H3K4me3 marked promoters across all cell types results in a tree in agreement with the known developmental relationships between cell types. **d** The average number of H3K4me3 detected peaks at the promoters is highly consistent across all the cell types. **e** The average H3K4me3 signal at common peaks across all cell types is highly variant, with BMDMs showing the strongest signal. **f** Hierarchical clustering of RNAPII (8WG16) binding is closely correlated with the H3K4me3 tree, rather than the expression tree. **g** The average number of RNAPII peaks at the promoters is consistent across cell types, however less than in H3K4me3 marked promoters. **h** The average RNAPII signal at common peaks at the promoters is highly variant with ESCs displaying the strongest signal. **i** Hierarchical clustering of H3K27me3 marked promoters across all cell types results in a tree where only the relationships of MBs and MTs are reconstituted. **j** The average number of H3K27me3 peaks at the promoters is variable across the cell types, with B cells showing the largest number of detected peaks in all cell types. **k** The average H3K27me3 signal at common peaks is highly variant across cell types with MEFs showing the strongest signal

To study the interplay between epigenetic and transcription control across cell types, we collected ChIP-sequencing data for two chromatin modifications (activating – H3K4me3 and silencing – H3K27me3) and RNA polymerase II (8WG16) as well as expression data (RNA sequencing) from eight cell types, including ES cells, in mouse (see Methods). The gene expression quantified at 22,179 GENCODE.vM4 [29] promoters (after discarding promoters with no signal for any mark or expression in all cell types, see Methods) showed very low variation in the number of expressed promoters (FPKM> 1, Additional file 1: Figure S1) across cell types (11,178 ± 396). The hierarchical clustering of cell types using expression data agreed with the known developmental relationships across the cell types (Fig. 1a) with the three hematopoietic cell types (B cells, bone-marrow derived macrophages (BMDMs) and dendritic cells (DCS)) clustered together while two progenitor cell types (progenitor motor neurons (PMNs) and ESCs) grouped together. Mouse embryonic fibroblasts (MEFs) clustered with Myoblasts (MBs) and Myotubes (MTs). Interestingly, gene expression signal calculated only at the promoters (see Methods) using RNA-seq data did not recapitulate known developmental hierarchy (Fig. 1b and Additional file 1: Figure S2).

We then determined H3K4me3, H3K27me3 and RNA polymerase II (RNAPII) signal at the promoters across eight cell types by calling peaks in each sample using SICER [30] and selecting the peaks within the 5 kb region around the Transcription Start Site (TSS) of the 22,179 genes. Though the number of H3K4me3 marked promoters across cell types (15,686 ± 804) varied more than the number of expressed genes across cell types, H3K4me3 modification was largely consistent with gene expression (Additional file 1: Table S4) at the promoters across 8 cell types. Accordingly, the hierarchical tree of H3K4me3 peaks at promoters across 8 cell types (Fig. 1c) was largely in agreement with the one obtained using expression data (Fig. 1a) apart from H3K4me3 profiles closely clustering MEFS with PMNs and ESCs. We confirmed this was not due to technical issues such as peaks calling bias across samples (Fig. 1d). For example, BMDMs and B cells clustered together despite a high signal variability at common H3K4me3 peaks found across all cell types (Fig. 1e). To study the dynamics of H3K4me3 modification between cell types, we used a maximum parsimony based approach (see Methods). Maximum parsimony approach predicts the chromatin modification status at each intermediate node of a tree by allowing minimum number of epigenetic changes within the tree [31, 32]. We noted that over 70% of promoters retain H3K4me3 modification across cell types (Additional file 1: Figure S3).

The hierarchical clustering of RNAPII (8WG16) modification at the promoters was not as consistent with the expression as the H3K4me3 (Fig. 1f). Notably, the number of RNAPII marked promoters varied highly across cell types (13,241 ± 2197) where BMDMs had over 16,000 RNAPII occupied promoters while DCs and B cells had only about 10,000 RNAPII occupied promoters (Additional file 1: Figure S5). This might be one of the reasons that DCs and B cells clustered together in the RNAPII hierarchical tree (Fig. 1f). Importantly, MEFs clustered together with PMNs and ESCs, similar to H3K4me3 (Fig. 1c). We tested the technical variability between samples by calculating average RNAPII peak strength in each cell type across common peaks (Fig. 1h). The RNAPII peak strength showed low correlation with the number of RNAPII peaks (Fig. 1g). The parsimony tree using RNAPII peaks at promoters demonstrated that RNAPII peaks were shared to a lesser extent, around 60%, than H3K4me3 between cell types (Additional file 1: Figure S4).

The number of H3K27me3 marked promoters varied even more across cell types (5648 ± 2405). B cells, BMDMS and MEFS had more than twice the promoters marked with H3K27me3 (~ 8000) as in PMNS, MBs and MTs (~ 3000). The hierarchical tree of H3K27me3 at promoters across cell types did not fully agree with expression data, as MEFS clustered with B cells and BMDMs, while DCs clustered with progenitor cells (Fig. 1i). We verified that this variability is not solely due to technical reasons by calculating average number of detected peaks across all promoters (Fig. 1j) and average H3K27me3 signal at common peaks in each cell type (Fig. 1k). In the H3K27me3 parsimony tree, only about 16% of H3K27me3 promoters were shared across all cell types (Additional file 1: Figure S5).

Bivalent promoters are marked with both repressing (H3K27me3) and activating (H3K4me3) modifications [14, 15]. We sub-classified H3K27me3 promoters into bivalent and H3K27me3-only promoters based on presence or absence of H3K4me3 modification at the same promoter in the same cell type. Over 90% of H3K27me3 promoters in ESCs and PMNS were bivalent while only about 60% of H3K27me3 promoters in B cells were bivalent (Additional file 1: Table S4). Interestingly, about half of bivalent promoters in ES cells were shared across cell types (Additional file 1: Figure S6) and were enriched for pattern specification process (*P* value < 10^− 8^) and developmental protein (*P* value < 10^− 15^).

Taken together, hierarchical clustering of H3K4me3 peaks at promoters agreed the most while H3K27me3 peaks at promoters agreed the least with known developmental hierarchies among eight cell types.

### Cataloguing major epigenetic and expression profiles at promoters across cell types

**Fig. 2:**
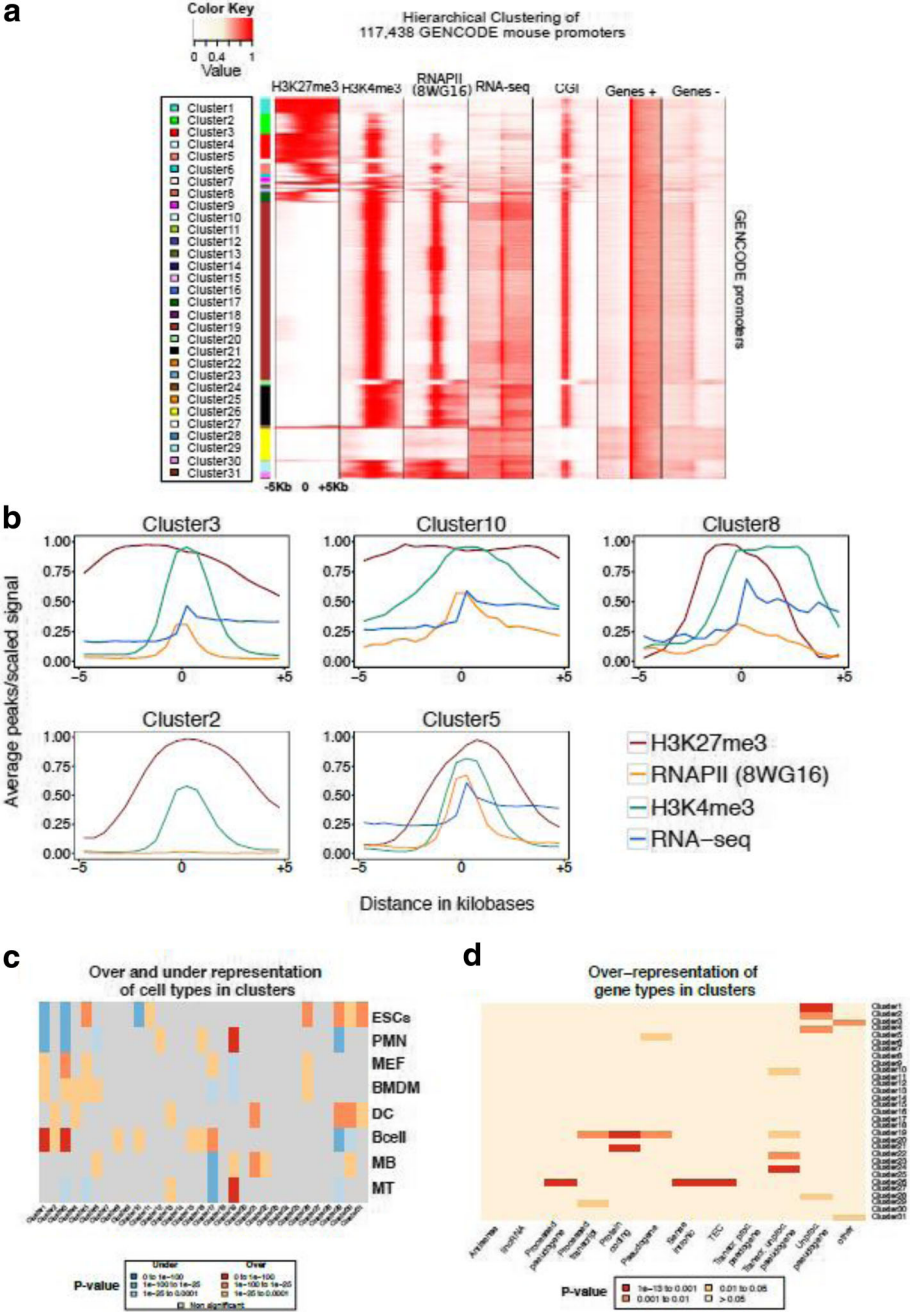
Epigenetic and expression profiles for 31 distinct clusters and their characterisation. **a** Hierarchical clustering of the profiles of H3K27me3 (peaks), H3K4me3 (peaks), RNAPII (peaks) and expression signal (reads per million) across 117,438 distinct gene promoter-cell type pairs. 31 clusters of distinct signatures were detected. **b** Average number of peaks /Average RNA-seq signal at representative clusters from 31 clusters, displaying divergent epigenetic and transcription profiles. **c** Under and over-representation of cell types in each cluster (significance was assessed with hypergeometric test). **d** Under and over-representation of gene types per cluster (significance was assessed with hypergeometric test)

To investigate the major patterns of chromatin and expression at promoters across cell types, we clustered H3K4me3, H3K27me3 and RNAPII peaks as well as RNA-seq signal at 22,179 GENCODE.vM4gene promoters in 8 cell types. Promoters occupied by RNAPII can be active and paused depending upon whether the RNAPII signal is more enriched at the core promoter than in the gene body [23, 24]. To capture such functionally relevant features of chromatin modifications, we defined a wide window (±5 kb) around the TSS at each promoter in a given cell type resulting into a total of 117,438 promoter-cell types (see Methods). We clustered promoter-cell types by hierarchical clustering using the Euclidean distance as a distance measure (see Methods), resulting in 31 clusters with distinct patterns across four data types (Figs. 2a and Additional file 1: Figure S7–37). The number of promoter-cell types in each cluster varied largely across clusters. Cluster 19 consisted over 54,000 promoter-cell types while cluster 8 consisted of only 105 promoter-cell types (Additional file 1: Table S5). H3K4me3 and RNAPII modifications largely overlapped with expressed promoters (Fig. 2a). The majority of H3K27me3 marked promoters also had strong H3K4me3 modification.

Multiple clusters were classified as bivalent, i.e. marked simultaneously with H3K27me3 and H3K4me3 modifications. Transcriptionally active bivalent clusters tended to have wide H3K27me3 and were grouped according to different levels of expression at the promoter (Fig. 2b). Lowly expressed bivalent clusters showed either wide (Clusters 10 and 3) or narrow (clusters 2 and 5) H3K27me3 pattern at the promoter. Bivalent-wide-H3K27me3 cluster 10 was enriched for ‘pattern specification process’ (*P* value < 10^− 30^) and ‘Embryonic morphogenesis’ (*P* value < 10^− 30^) and cluster 3 was enriched for ‘nervous system development’ (*P* value < 10^− 30^). On the other hand, bivalent-narrow-H3K27me3 cluster 2 showed high enrichment for ‘cell-cell signalling’ (*P* value < 10^− 30^) and cluster 5 was highly enriched for genes involved in ‘signalling’ (*P* value < 10^− 20^).

We have previously noted that bivalent promoters were enriched while H3K27me3-only promoters were deprived of CpG islands in mouse and human ESCs [33]. Average CpG density at promoters in all clusters revealed that active and bivalent clusters were enriched for CpG islands (over 80% promoters with CpG island, Additional file 1: Figure S7C). The H3K27me3-only clusters 1 and 4 indeed showed lower CpG islands (less than 50% promoters with CpG island, Additional file 1: Figure S7C) and low mean CpG densities (Additional file 1: Figure S7A and B) albeit much higher than in mouse ES cells.

We then investigated if particular cell types were over or under-represented in the clusters by hypergeometric testing after correcting for cell type specific differences (see Methods). In over half of the clusters, all cell types were equally represented (Fig. 2c). ESCs were underrepresented while B cells were over-represented in H3K27me3-only clusters 1 and 4 (Fig. 2c). Bivalent clusters were initially thought to be exclusive to ESCs [34] but were later on found in mature cell types but in fewer promoters than ESCs [15, 16]. Surprisingly, ESCs were not over-represented in most bivalent clusters (Fig. 2c). Mono-allelic expression can be one of likely sources of what is detected as bivalent chromatin. Indeed bivalent gene clusters showed higher fraction of mono-allelically expressed genes than active gene clusters (Additional file 1: Figure S8). Interestingly H3K27me3-only clusters 1 and 4 showed a very high overlap with mono-allelically expressed genes as well (Additional file 1: Figure S8).

To identify clusters with shared epigenetic and transcription profiles across cell types, we calculated the ratio of unique promoters to all promoters in each cluster (Additional file 1: Table S5). This ratio was the lowest for cluster 19 (0.24, Additional file 1: Table S5) with expressed genes enriched for ‘cellular macromolecule catabolic process’ (*P* value < 10^− 30^). Five other clusters (active clusters 20, 21 and 26 and bivalent clusters 3 and 24) contained promoter profiles conserved across cell types (Additional file 1: Table S5). We further studied the extent of conservation of genes in each cluster across cell types. Promoter profiles of active clusters showed higher conservation across cell types than bivalent clusters. About 15% of the promoters in ES cells in bivalent cluster 3 remained bivalent in other cell types. In contrast, only about 3% of promoters in ES cells in bivalent cluster 5 remained bivalent in other cell types. Bivalent cluster 3 with wide H3K27me3 and high RNAPII was associated with positive regulation of transcription (*P* value < 10^− 2^), somatic stem cell population maintenance (*P* value < 10^− 6^) and regulation of transcription for RNAPII promoter in response to stress (*P* value < 10^− 4^) while bivalent cluster 5 with narrow H3K27me3 was enriched for functions such as regulation of cytokine biosynthetic process (*P* value < 10^− 2^), liver development (*P* value < 10^− 2^) and regulation of myeloid cell differentiation (*P* value < 10^− 2^). This is in agreement with our previous results where bivalent promoters separated in clusters with different levels of RNAPII and variable expression [33].

We calculated enrichments for GENCODE-defined gene types in each cluster (Fig. 2d). Clusters 19 and 21 with high levels of H3K4me3 and RNAPII and high expression were highly enriched for protein coding genes (hypergeometric test, *P* value < 0.001). Cluster 26 was enriched for processed pseudogene and sense intronic RNA (P value < 0.0001, Fig. 2d). Many genes belonged to cluster 26 in multiple cell types and showed functional enrichment for G-protein coupled receptor signalling pattern (*P* value < 10^− 10^) and sensory perception of chemical stimulus (*P* value < 10^− 7^).

Taken together, the chromatin and expression profiles of promoters in eight cell types formed clusters enriched for specific functional properties.

### Transcription factor binding and motif enrichment across the clusters

**Fig. 3:**
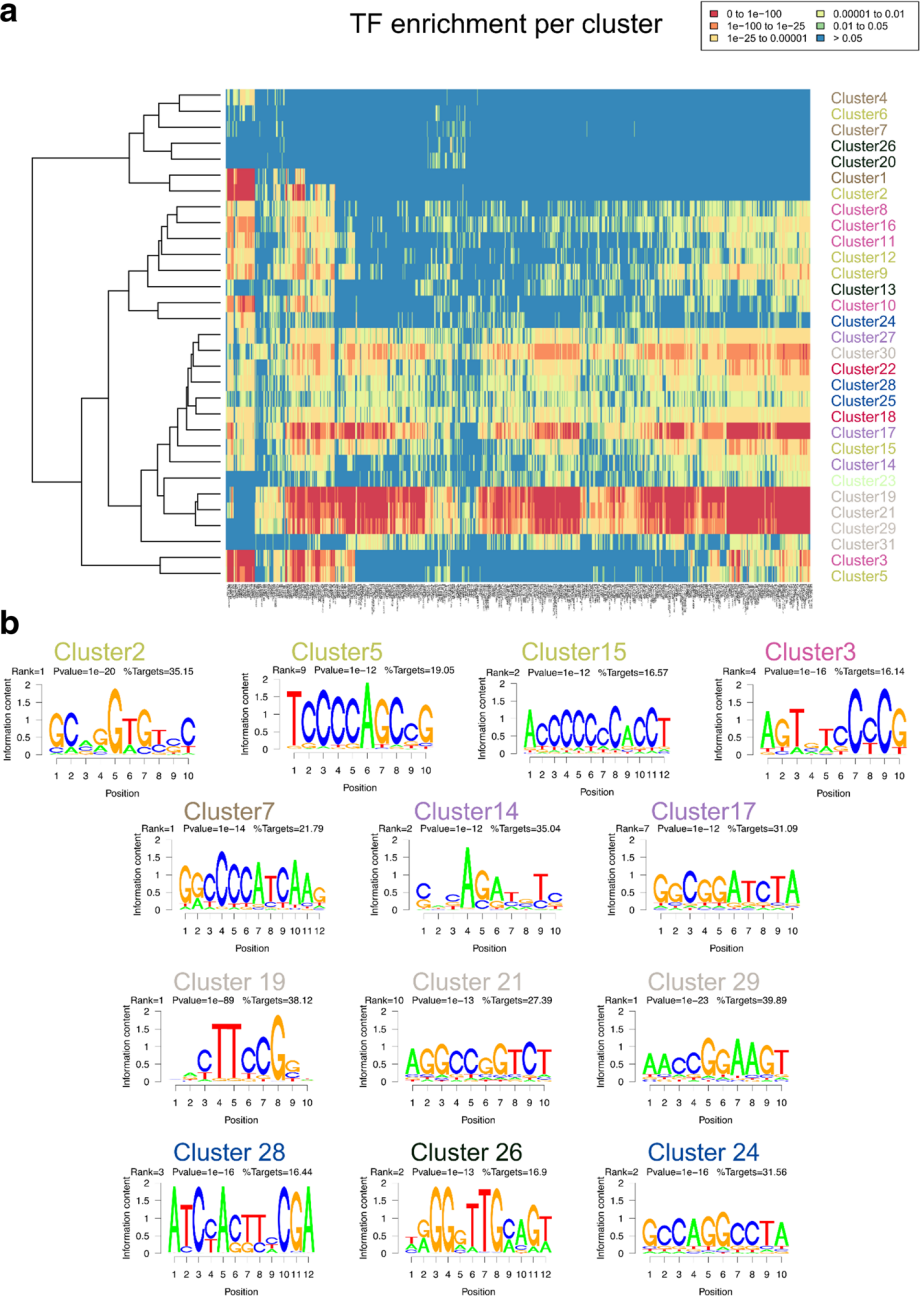
Transcription factor binding and motif enrichment across the clusters. **a** Transcription-related factor binding enrichment using the CODEX (Sánchez-Castillo et al. 2015) ChIP-seq data compendium (see Methods). All clusters were significantly enriched (hypergeometric test - *P* value < 0.01) for at least one factor. **b** Thirteen clusters showed at least one de-novo motif enrichment with more than 15% of targets and *P* value < 10^− 10^

To investigate whether clusters were enriched for binding of specific transcription and epigenetic controllers, we calculated binding enrichment using the CODEX [35] ChIP sequencing data compendium (see Methods). All clusters were significantly enriched (hypergeometric test - *P* value < 0.001) for at least one factor (Fig. 3a). Highly-active clusters 19, 21 and 29 were enriched for binding of many transcription and epigenetic controllers. H3K27me3-only cluster 1 and bivalent cluster 2 were both highly enriched for Polycomb (Suz12, Ezh2, Rnf2, Mtf2 and Ring1b) as well as Kdm2b, Notch1 and Klf2 binding. Bivalent cluster 2 was additionally enriched for binding of Hdac2, Ldb1 and Foxa2.

**Fig. 4:**
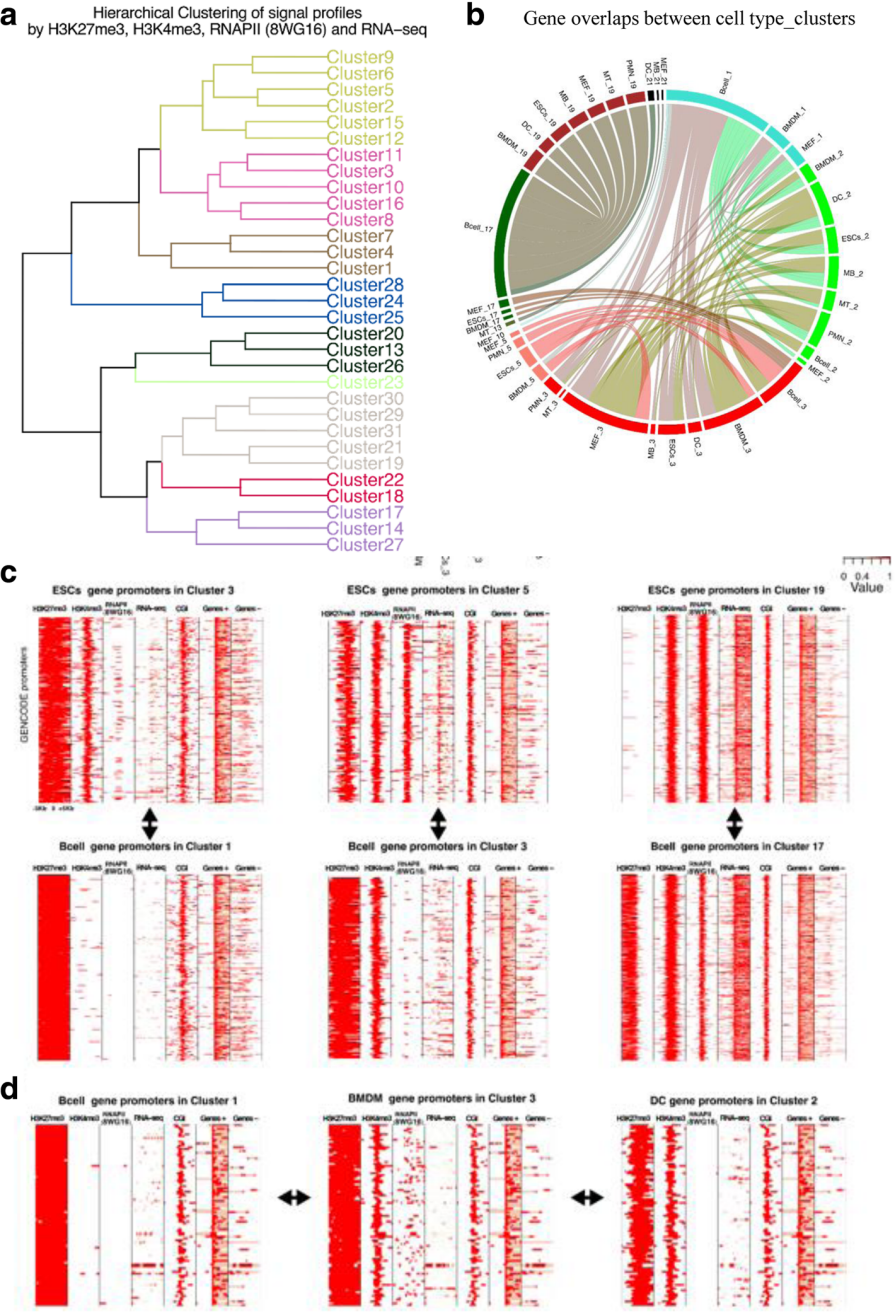
Promoter dynamic across cell types and chromatin states. **a** Hierarchical clustering of the average profile signals across clusters results in the identification of 9 major profile sub-groups. **b** Significant chromatin state transitions across cell types and clusters. Four major chromatin state changes across cell type pairs emerged, namely H3K27me3-only <− > bivalent-wide-H3K27me3, H3K27me3-only <− > bivalent-narrow-H3K27me3, bivalent-narrow-H3K27me3 < −> bivalent-active and bivalent-active <− > highly-active. **c** Bivalent-wide-H3K27me3 cluster 3 promoters in ESCs overlapped highly H3K27me3-only cluster 1 promoters in B cells and were enriched for ‘pattern specification process’ (*P* value < 10^− 30^). Bivalent-narrow-H3K27me3 cluster 5 promoters in ESCs overlapped highly with bivalent-wide-H3K27me3 cluster 3 promoters in B cells and were enriched for ‘Nervous system development’ (*P* value < 10^− 30^). Highly-active cluster 19 promoters in ESCs overlapped highly with boundary-H3K27me3-active cluster 17 promoters in B cells and were enriched for ‘ncRNA metabolic process’ (*P* value < 1.7.10^− 23^). **d** Significant sets of genes overlapping in 3 distinct cell types and clusters. 98 genes enriched for ‘cell fate commitment’ (*P* value < 3.2.10^− 7^) were present in B cells in cluster 1, in DCS in cluster 2 and in BMDMS in cluster 3

The similarities between the epigenetic and transcription profiles of clusters largely agreed with the similarities in their ChIP-seq binding, i.e. the hierarchical clustering of binding enrichment at each cluster promoter (Fig. 3a) resulted in the same sub-grouping as obtained using similarities between the profiles (Fig. 4a) for many clusters. For example, clusters 19, 21, 29 and 31 contained active promoters and showed enrichment for similar TFs. Two bivalent-wide-H3K27me3 clusters (11, and 16) were also clustered close to each other with significantly high enrichment for Polycomb components. Clusters 20 and 26 were significantly enriched for binding of similar TFs mostly involved in hematopoietic development including Eto2, Tal1, Lmo2 and Gata2 [36–38]. Nevertheless, there were few cases where divergent epigenetic profiles had similar transcription binding. For example, bivalent-wide-H3K27me3 cluster 3 and bivalent-narrow-H3K27me3 cluster 5 were enriched for binding of very similar factors despite the differences in H3K27me3 signal and RNAPII occupancy.

We further performed de-novo and known motif discovery for all clusters using HOMER [39]. Thirteen clusters showed at least one de-novo motif enrichment with more than 15% of targets and *P* value < 10^− 10^ (Fig. 3b). Bivalent clusters 2, 3, 5, and 15 were enriched for GC-rich motifs. Bivalent clusters 3 and 5 were enriched for a ‘TCCCC’ sequence motif, previously identified enriched at bivalent promoters in both mouse and human ESCs [33], while bivalent cluster 2 was enriched for a ‘GGTCT’ motif, previously identified as a consensus binding sequence for the *Drosophila melanogaster* Tbx20 T-box transcription factor homolog Midline [40]. GC-rich motifs were highly enriched in clusters with H3K27me3 modifications including clusters 7, 14 and 17. The promoter sequences of active clusters 19 and 29 were enriched for variants of a GABP motif. The motifs (CTTCCG, CCGGAA) enriched at the active promoters have previously been identified as the coding motifs i.e. motifs enriched in coding sequences [41]. Finally, cluster 24 was enriched for a GC-rich G-box like motif (‘GCCAGGCCT’), present in about a third of promoters in the cluster.

Taken together, clusters wit similar epigenetic profiles were enriched for binding of similar factors and many clusters were enriched for specific de-novo sequence motifs.

### Promoter dynamics across cell types and chromatin states

To study chromatin and transcriptional state transitions across cell types, we calculated the number of overlapping genes across cell types in individual clusters. The statistical significance of the number of promoters shared between cell types across clusters was calculated using hypergeometric test after correcting for the cell type bias of each cluster (see Methods). The majority of significant overlaps were between clusters with similar epigenetic and expression status represented by the hierarchical clustering of the signal profiles of H3K27me3, H3K4me3, polII and RNA-seq at promoters (Fig. 4a). For example, 632 genes belonged to cluster 19 in PMNS and to cluster 29 in ESCs. Though these genes are expressed in both cluster 19 and 29, they show a wide H3K4me3 and RNAPII signal upstream of TSS in ESCs while a sharp narrow H3K4me3 and RNAPII signal at the promoter in PMNS.

To focus on the major chromatin and transcriptional state transitions across cell types, we further grouped 31 clusters into 9 major sub-groups namely: i) bivalent-narrow-H3K27me3, ii) bivalent-wide-H3K27me3, iii) H3K27me3-only, iv) bivalent-wide-active, v) wide-active, vi) antisense-active, vii) highly-active, viii) bivalent-highly-active, ix) boundary-H3K27me3-active, based on hierarchical clustering (Fig. 4a). There were four major significant overlaps across cluster sub-groups (Fig. 4b) with chromatin state changes between cell types namely H3K27me3-only ↔ bivalent-wide-H3K27me3, H3K27me3-only ↔ bivalent-narrow-H3K27me3, bivalent-narrow-H3K27me3 ↔ bivalent active and bivalent active ↔ highly-active. H3K27me3-only promoters in B cells were either bivalent-narrow-H3K27me3 or bivalent-wide-H3K27me3 in many other cell types. Similarly, bivalent active promoters in B cells were active in many other cell types. Bivalent-wide-H3K27me3 cluster 3 promoters in ESCs overlapped highly H3K27me3-only cluster 1 promoters in B cells and were enriched for ‘pattern specification process’ (*P* value < 10^− 30^) (Fig. 4c). Bivalent-narrow-H3K27me3 cluster 5 promoters in ESCs overlapped highly with bivalent-wide-H3K27me3 cluster 3 promoters in B cells and were enriched for ‘Nervous system development’ (*P* value < 10^− 30^) (Fig. 4c). Highly-active cluster 19 promoters in ESCs overlapped highly with boundary-H3K27me3-active cluster 17 promoters in B cells and were enriched for ‘ncRNA metabolic process’ (*P* value < 10^− 23^) (Fig. 4c). To exclude the possibility that the aberrant H3K27me3 modification at the promoters in B cells compared to the other cell types was due to technical issue of the sample, we replaced the H3K27me3 sample in B cells with another H3K27me3 ChIP-seq replicate in B cells from the same study and found the same result (Additional file 1: Figure S9A and S9B), despite overall decrease in the H3K27me3 signal in the alternative sample.

We further calculated statistical significance for the promoters belonging to three different clusters in three cell types. As expected most of the significant cluster triplets consisted of promoters in clusters 1 in B cells present in cluster 2 and cluster 3 in other two cell types. For example, 98 genes enriched for ‘cell fate commitment’ (*P* value < 10^− 6^) were present in B cells in cluster 1, in DCS in cluster 2 and in BMDMs in cluster 3 (Fig. 4d).

Taken together, we noted significant patterns of epigenetic dynamics across cell types predominantly between 6 clusters (clusters 1, 2, 3, 17, 19 and 21). Importantly, the major epigenetic state dynamics across cell types was not reflected at the expression level. Importantly, bivalent promoters in ESCs did not overall become either active or repressed in other cell types, contrary to postulations in literature [15, 42].

### RNAPII pausing across clusters and cell types

**Fig. 5:**
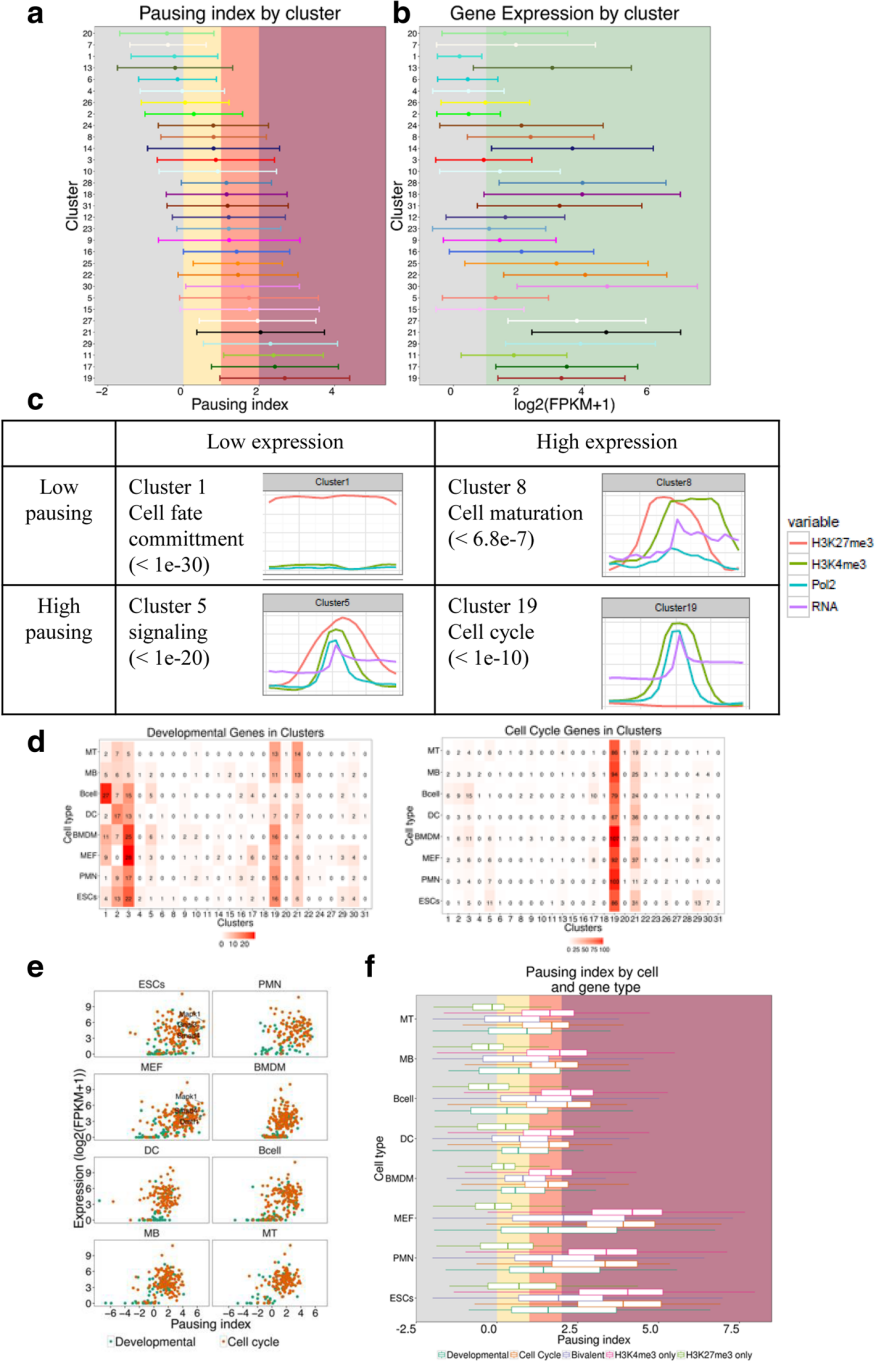
RNAPII pausing across clusters and cell types. **a** Distribution of pausing indices across gene promoters in all 31 clusters in our study. Clusters are ordered according to their mean pausing index. The colours in the background denote the level of RNAPII pausing - grey: no pausing, yellow: low pausing, red: mid pausing, dark red: high pausing. **b** Distribution of expression levels (log2(FPKM+ 1)) of genes in each cluster. The clusters arranged in the same order as in Fig. 5a. The colour background denotes a threshold on expression (log2(FPKM+ 1) =1) as defined in Additional file 1: Figure S1 – grey: lowly or not expressed, green: expressed. **c** Four representative clusters displaying four combinations of RNAPII pausing and gene expression. **d** Distribution of developmental (GO:0045165, GO:0048864, GO:0007498) and cell cycle (GO:0007049) genes across clusters. **e** RNAPII pausing vs expression for developmental and cell cycle genes (see Methods) across cell types. **f** Mean pausing indices for diverse gene sets in each cell type. The gene sets were developmental, cell-cycle as well as bivalent, H3K4me3-only and H3K27me3-only genes in ES cells

We calculated the RNAPII pausing index for all genes in clusters defined as the ratio of RNAPII signal at the core promoter to RNAPII signal within the gene body (see Methods) [43]. We ordered the clusters according to their respective mean pausing index (Fig. 5a). The majority of active clusters had relatively high pausing index while H3K27me3-only clusters had very low pausing and low mean expression (Fig. 5a, b). Specifically, active clusters 17, 19, and 29 had the highest pausing indices (more than 2, in dark red background in Fig. 5a) compared to the H3K27me3-only clusters 1, 4, and 7 had very low or no pausing at all (in yellow/grey background, Fig. 5a). Though there was a moderate correlation between the average pausing index and average mRNA expression levels across clusters (Pearson’s correlation coefficient 0.53), the two did not correlate with each other in every cluster (Fig. 5c). Genes in bivalent clusters were expressed at low levels but showed variable pausing index. Clusters enriched for cell fate commitment and maturation showed low pausing while clusters enriched for signalling and cell cycle genes were highly paused (Fig. 5c). This is in agreement with a recent publication demonstrating that high pausing index is more associated with highly expressed genes involved in cell cycle regulation rather than bivalent developmental regulators [44].

As RNAPII pausing is shown to be involved in the transcription of genes involved in both development and cell cycle [45–47]**,** we calculated the pausing indices for genes in the functional categories of cell cycle and developmental genes (see Methods). The developmental genes mostly belonged to H3K27me3-only cluster 1, bivalent clusters 2 and 3 and active clusters 19 and 21 (Fig. 5d) while cell cycle genes were found mostly in active clusters 19 and 21 (Fig. 5d). Cell cycle genes showed high levels of RNAPII pausing in progenitors and mid pausing levels in committed cell types. RNAPII pausing in developmental genes also decreased from mid to low levels from progenitors to committed cell types (Fig. 5e). The average pausing index for expressed genes in each cell type, irrespective of clusters showed that indeed the cell types with high multipotency potential (ESCs, PMNS and MEFS) had a high mean pausing index (Fig. 5f). Bivalent genes in ES cells showed mean pausing index similar to developmental genes while active genes (H3K4me3-only) showed mean pausing index similar to cell cycle genes in each cell type (Fig. 5f). Williams et al. (2015) proposed that RNAPII pausing in ESCs facilitates maintainance of self-renewal potential. We calculated pausing index for Pro-pluripotency, Pro-differentiation and ES signalling groups from KEGG pathways [48] in all cell types. Self-renewal (Pro-pluripotency) genes exhibited extremely high RNAPII pausing (pausing index > 4) and high expression is ESCs, PMNs and MEFS, but not in the other cell types (data not shown).

In conclusion, RNAPII pausing is higher at active genes than at bivalent genes within a cell type, and is higher in progenitor cell types compared to differentiated cell types. Pro-pluripotency and cell cycle genes are highly paused and highly expressed in ESCs, PMNS and MEFS. This supports that RNAPII pausing might assist cells retain their pluripotent characteristics.

## Discussion

We integrated epigenetic marks (H3K4me3, H3K27me3), RNAPII (8WG16) binding and expression (RNA-seq) in eight mouse cell types of variable developmental potential. Hierarchical clustering of histone marks at promoters differed from that of the expression data. Specifically, MEFS were closer to MBs and MTs in the expression hierarchical tree (Fig. 1a) whereas they clustered with progenitor cells (ESCs, PMNS) in the H3K4me3 and RNAPII trees (Fig. 1c and f). The H3K27me3 tree was greatly discordant with the known developmental relationships between cell types. This could be partly due to a large variation in the average number of detected peaks across cell types (Fig. 1j). The differences in the number of peaks were not reflected in the signal at the common peaks (Fig. 1k), suggesting that there might be a real biological difference between H3K27me3 occupancy at promoters across cell types.

The clustering of profiles of silenced (H3K27me3) and predominantly active (H3K4me3, RNAPII and RNA-seq) signals across promoters for all the cell types resulted in 31 distinct clusters. Genes in active clusters (19, 20, and 21) were expressed across many cell types compared to some bivalent clusters (3 and 24) (Additional file 1: Table S5). Bivalent clusters formed two main clusters (3 and 5) with different H3K27me3 and RNAPII profiles. Bivalent cluster 3 with wide H3K27me3 and high RNAPII was enriched for genes involved in transcription control while bivalent cluster 5 with narrow H3K27me3 was enriched for genes in development and differentiation. This is in agreement with our previous results where HC bivalent promoters form separate clusters, with different levels of RNAPII and variable expression [33]. Furthermore, cluster 3 was uniquely enriched for binding of activating factors such as Nanog, Oct4 and p300 (Additional file 1: Table S6). These results suggest that genes in cluster 3 are possibly directly affected by pluripotency and signalling factors (Additional file 1: Table S6) with RNAPII mark present at their promoters [44]. In contrast, bivalent genes in cluster 5 are more tissue-specific, and show higher levels of expression possibly due to multi-lineage priming [49].

Chromatin state transitioning between cell types were predominantly gain/loss of either H3K4me3 (H3K27me3-only ↔ bivalent) or H3K27me3 (active ↔ boundaryH3K27me3active) (Fig. 3a). Importantly, these chromatin state transitions were mostly not accompanied by expression changes. Most epigenetic transitions were between B cells and other cell types. Most bivalent genes in cluster 3 in other cell types belonged to H3K27me3-only cluster 1 in B cells. This is unlikely to be an experimental artefact as we validated the results with an independent dataset. Given that cluster 1 was highly enriched for monoallelically expressed genes, it proposes that bivalency in one cell type might be a mechanism to achieve monoallelic expression in other cell types. Indeed, random monoallelically accessible elements in neural progenitor cells were biallelically accessible in embryonic stem cells, but premarked with bivalent histone modifications; one allele was silenced during differentiation [50].

We used ChIP-seq data for RNAPII (8WG16), which efficiently reproduces results from global run-on sequencing (GRO-seq) [51] data to calculate RNAPII pausing [44]. RNAPII pausing index largely correlated with the expression across clusters: the majority of active clusters exhibited a highly pausing, highly expressed, bivalent clusters had mid-pausing levels, while H3K27me3-only clusters had low or no pausing (Fig. 5a). Importantly, ESCs, MEFs and PMNS had higher pausing indices than other cell types in this study (Fig. 5e). Also, MEFs showing high PolII and H3K4me3 profile with ESCs might help them better retain their ES-like characteristics than other cell types of similar developmental hierarchy [52], and therefore likely a good candidate cell type for induced pluripotent stem cells (iPSCs) reprogramming experiments [53].

A recent study by Liu J et al. [54] looked at the developmental changes of the bivalent chromatin marks and paused Pol II during neuronal differentiation in the mouse brain, and noted in agreement to our study that genes involved in cell cycle and other catabolic processes show very high RNA pol II pausing. Unlike our study, Liu J et al. [54] noted that bivalent marks primed neuronal specification genes for activation during differentiation. One of the possible reasons for the discrepancy might be that the data collected for eight cell types from public domain in this study does not have a well-defined developmental trajectory as during neuronal differentiation.

## Conclusion

We integrated epigenetic and transcription marks and gene expression at promoters of eight cell types of various developmental potential. We grouped promoters in distinct clusters based on their epigenetic and transcription profile and explored diverse functionalities of these clusters. Bivalent clusters did not show a high RNAPII pausing, highlighting that RNAPII pausing is mainly associated with active genes, and not bivalent genes. This is in agreement with the fact that Pol II pausing marks gene silenced after, or ready for, expression burst [55]. Finally, we showed that MEFs, PMNs and ESCs have higher overall pausing than other cell types studied (Fig. 5e).

## Methods

### ChIP-seq data collection and processing

ChIP-seq datasets for H3K4me3, H3K27me3 and RNAPII (8WG16) were collected in fastq format from Gene Expression Omnibus (GEO) [56] for eight mouse cell types (ESC, PMNS, MEFS, BMDMS, DCS, B cell, MBs, MTs) (Additional file 1: Table S1). Alignment of reads was done using Bowtie 2 using the mm10 reference genome and the default parameters [57]. SAM to BAM conversion of the aligned files was done using the SAMtools pipeline [58]. Total number of reads aligned to the genome is shown at Additional file 1: Table S2. The bam files that belonged to the same experiment (technical replicates) were merged into a single bam file.

### Peak calling

SICER [30] was used to detect peaks for the histone marks (H3K4me3 and H3K27me3). Input controls were not used, as they were not available for some cell types. Specific parameters were defined for H3K4me3, *window = 200* and *gap size = 200*. For H3K27me3 on the other hand, *window = 200* and *gap size = 2 × 300*, since this mark covers wider chromatin domains. The rest of the parameters (same for H3K4me3 and H3K27me3) were *effective genome fraction = 0.7*, *redundancy threshold = 1*, *fragment size = 150* and *E-value = 100*. MACS [59] was used for the detection of peaks for the RNAPII samples, using the default parameters and no input. The number of peaks detected for each sample are shown at Additional file 1: Table S2.

### RNA-seq data collection and processing

Corresponding RNA-seq datasets were collected from GEO [56] in fastq format for all of the eight cell types mentioned previously (Additional file 1: Table S3). Alignment was done with TopHat 2.0.9 [60] using mm10 as reference genome and the GENCODE.vM4 (Harrow et al., 2012) as annotation file. Expression values for each cell type were calculated following the Cufflinks 2.2.1 [61] pipeline. The aligned reads were converted to expression values using the *cuffquant* command with *library-type = fr-unstranded*. A bam file was created for each of the samples. Gene expression values (FPKM values) for all cell types was generated using the *cuffnorm* command with the default library normalization method (*geometric*).

### Hierarchical trees for histone marks and gene expression

GENCODE.vM4 [62] was the chosen annotation for the creation of custom promoter regions (22,179 unique genes with gene length > 300 bp). The promoter BED file was created by taking the − 5 kb, + 5 kb area around the TSSs of GENCODE genes. Peak BED files were intersected with the custom promoter file using the *intersectBED* command from the BEDtools suite [63]. The intersected peak-promoter files from all cell types were merged into one file, where each row was representing a gene promoter. In the columns, eight for each cell type, binary values of 0 or 1, would represent the absence or existence respectively, of a peak at that promoter for that cell type. For the hierarchical tree of gene expression, the output matrix from *cuffnorm* command was used. Hierarchical clustering was performed using the *hclust* function from the *fastcluster* package in R [64]. The *Euclidean* distance of the columns of each matrix (cell types) was used as a dissimilarity matrix and the method chosen was *complete*.

### Clustering of gene promoters across cell types

We loaded the peak files in R for all the ChIP-seq datasets and converted them to GRanges objects. The bam files for each RNA-seq sample were also loaded in R creating custom coverage files with the *GRcoverageInbins* function (as object we used the promoter file (32,840 regions) converting it to GRanges, *Nnorm* = TRUE, *Snorm* = FALSE, *Nbins* = 20) from the *compEpiTools* package [65] in Bioconductor [66]. For each of the cell types, we subsequently created a combined matrix of histone marks, RNAPII, RNA-seq coverage (normalized by library size in each cell type), CpG island regions (from UCSC) and gene annotation for sense and antisense transcripts (Gencode.vM4). Using the *heatmapData* function from *compEpiTools* [65] we created a 140 column matrix (20 bins for each of the features) where the first 20 columns were representing the H3K27me3 peaks, columns 21–40 were H3K4me3 peaks, columns 41–60 were RNAPII peaks, columns 61–80 were RNA-seq coverage, columns 81–100 were CpG islands, columns 100–120 were sense transcript annotation and columns were 120–140 antisense transcript annotation. RNA-seq coverage was log2-scaled and transformed (to obtain values only in the (0,1) range) for each cell type separately.

We combined the matrices from all the cell types to acquire an initial matrix of 32,840*8 = 262,720 rows. Each row had a distinctive name of the ensembl gene id and the cell type it belonged to. We subsequently discarded the rows where more than 80% of the columns (only columns 1 to 80 were considered) had a zero value. This resulted in a matrix of 117,438 rows where each gene promoter was found in at least one cell type.

Hierarchical clustering of the gene promoter matrix was performed using the *hclust* function from the *fastcluster* package in R [64] using *Euclidean* distance. Only the histone marks, RNAPII and expression values were taken into account, and CGI and gene annotations were not. After inspection of the initial clustering, through heatmap visualisation, we cut the resulting tree in groups using the *cutree* function from *stats* package in R [67] and *k* = 60. The high number of groups specified facilitated the detection of groups that had small number of genes, but presented a highly unique pattern of marks or expression.

We created a custom function in R to merge the clusters with similar patterns. The central function incorporated in our function was *clusterSim* (method = *“centroid”*) from the *flexclust* package in R [68]. *clusterSim* computed the pairwise distances between all centroids of the 60 groups and scaled them between (0,1). The similarity value was then given by subtracting the distance from 1. We merged clusters with similarity values were over the 99th quantile of the similarity values distribution for all pairwise comparisons. The newly merged clusters along with the ones that were not similar with any other cluster were renamed. Then clusters with less than 100 genes were not considered for further analysis. We defined the peaks as ‘wide’ if they spanned more than 3 kb, otherwise ‘narrow’.

The clusters were visualised with the *heatmap.2* function from the *gplots* package in R [69]. The final matrix contained 116,741 gene promoters with 22,179 unique genes.

### Over and under representation of cell types in clusters

Significance of over or under representation of each cell type in each cluster was assessed using the hypergeometric test in R (*phyper*) from *stats* package. Since the cell types were not equally represented in the total population, we calculated the test using normalised values for the number of genes across clusters. The number of genes in a cluster for a one cell type were divided by the total number of genes for that cell type and then multiplied by 10,000, resulting in a matrix where virtually the total number of genes would be 8 (cell types) *10,000 = 80,000.

### Functional enrichment analysis

Gene Ontology (GO) enrichment analysis was done using the *topGO* package [70] in Bioconductor [66] and the statistical test used to quantify the significance of the GO terms was *fisher’s exact test*.

### Maximum parsimony trees

For each cluster gene list, we created a matrix where the rows where the unique ensembl gene ids of that cluster and the columns where the names of the 8 cell types. If a gene was found in that cluster for a particular cell type a value of 1 was denoted, else the value was set to 0. The downstream analysis was conducted for each cluster separately and the binary matrices were the inputs for the next step where we used the *ape* package in R [71]. First we calculated the pairwise distances between the genes in the matrix using the *dist.gene* function. The neighbour joining tree estimation [72, 73] was performed with the *nj* function using as input the result from the right previous step. Finally, the reconstruction of the most parsimonious ancestral states [31, 32] was done using the *MPR* function were we used as inputs the initial binary matrix, the resulting tree from the previous step, the “ESC” cell type as *outgroup,* with only the lower values of the reconstructed sets for each ancestral node.

### Average profiles and profile similarities

We calculated the average values for the columns of the matrix representing the 10 kb region around the TSS, for the histone marks, RNAPII and RNA-seq of each cluster. This resulted in one row matrices containing, mean profiles for all the variables used for clustering of the promoters. We performed a hierarchical clustering for all the mean profile matrices using the *hclust* function from the *fastcluster* package in R [64]. The *Euclidean* distance of the rows was used as a dissimilarity matrix and the method chosen was *complete*.

### Gene overlap between clusters and cell types

We calculated the overlap of genes between pairwise combinations of “Cell type - Cluster” sets of genes. For example, the overlap of genes between Cluster 1 genes in B cells with Cluster 3 genes of ESCs. The significance of the overlap was assessed by the hypergeometric test in R (*phyper*) from *stats* package. As the genes were not equally represented in each cell type, thus the normalized number of genes was used. Gene overlaps with *P* value < 10^− 10^ and the number of overlapping genes was larger than 50 were considered significant. To visualize the interactions between the “Cell type - Cluster”, we used the *chordDiagram* function from the *circlize* package [74] in R. The size of the links is defined by the number of genes overlapping and the colour is a mix between the colours defining the clusters of interaction. We also calculated the overlap of genes across triplets of “Cell type - Cluster” sets of genes and kept only the interactions where more than 50 genes were overlapping.

### Transcription factor enrichment

We downloaded data from 683 ChIP-seq experiments of TFs in multiple mouse cell types from the CODEX database [35]. We calculated the overlap of the TF binding regions with the regions 1 kb around the TSS of each gene in each cluster. We used the function *countOverlaps* from the *GenomicRanges* package [75] in Bioconductor [66]. To assess the significance of the overlaps we used the hypergeometric test in R (*phyper*) from *stats* package.

### Motif enrichment

Using the unique ensembl gene ids from the gene promoters in each cluster, we used the *findMotifs.pl* command from the HOMER suite [39] and searched for known and de-novo motifs at the 1 kb areas flanking the TSSs. We selected de-novo motifs that were represented in more than 15% of the regions and a *P* value < 10^− 10^. Similarly, known motifs in more than 15% of the regions and a *P* value < 10^− 5^ were selected.

### RNAPII pausing index calculation

The RNAPII pausing index (travelling ratio) defined by Muse et al. 2007 was used.

$$ S={\mathit{\log}}_2\left(d\left({RNAPII}_{promoter}\right)\right)-{\mathit{\log}}_2\left(d\left({RNAPII}_{genebody}\right)\right) $$

It is the ratio of RNAPII read density at the promoter to the RNAPII read density in the gene body. *d* stands for the number of reads per nucleotide (nt) in the given region. The difference between the densities in log2 units equals to the ratio of fold enrichment in these regions, meaning a value of 1 would represent a 2-fold greater enrichment of RNAPII signal at the promoter region rather than in the gene body (Muse et al. 2007). We created two *GRanges* objects: 1) The promoter area ranging 600 bp around the TSS of the gene and 2) the gene body area ranging + 600 from the TSS until the Transcription Ending Site (TES) of the gene. Using the *GRcoverage* function (as objects we used the previously mentioned promoter and gene body files (22,179 regions), *Nnorm* = FALSE, *Snorm* = TRUE) from the *compEpiTools* package [65] in Bioconductor [66] we computed the read coverage at those regions for each cell type and gene in our clusters.

We calculated pausing indices for the genes categories: developmental, cell cycle, pro-pluripotency, pro-differentiation and ES signalling genes. Williams et al. 2015 developmental and cell cycle gene lists were used, whereas the signalling genes were obtained from KEGG pathways database [48]. For developmental genes the GO terms were: GO:0045165, GO:0048864, GO:0007498 and for cell cycle genes were: GO:0007049. We defined pro-pluripotency (stem cell population maintenance – GO:0019827, negative regulation of cell differentiation – GO:0045596), pro-differentiation (positive regulation of cell differentiation – GO:0045597) and ES-signalling genes (cytokine activity – GO:0005125, regulation of MAPK cascade – GO:0043408).

## Additional file

Additional file 1: Supplementary tables and figures for Mantsoki et al. 2018. (DOCX 1190 kb)

## Abbreviations

BMDM: bone-marrow derived macrophages
DC: Dendritic cell
ESC: Embryonic stem cell
iPSC: induced pluripotent stem cell
MB: Myoblasts
MEF: Mouse embryonic fibroblasts
MT: Myotubes
PMN: Progenitor motor neurons
RNAPII: RNA polymerase II

## References

[1] Reik W (2007). Stability and flexibility of epigenetic gene regulation in mammalian development. Nat Publ Group.

[2] Rivera CM, Ren B (2013). Mapping Human Epigenomes. Cell.

[3] Zhu J, Adli M, Zou JY, Verstappen G, Coyne M, Zhang X (2013). Genome-wide chromatin state transitions associated with developmental and environmental cues. Cell.

[4] Xie W, Schultz MD, Lister R, Hou Z, Rajagopal N, Ray P (2013). Epigenomic analysis of multilineage differentiation of human embryonic stem cells. Cell.

[5] Thurman RE, Rynes E, Humbert R, Vierstra J, Maurano MT, Haugen E (2012). The accessible chromatin landscape of the human genome. Nature.

[6] Maurano MT, Humbert R, Rynes E, Thurman RE, Haugen E, Wang H, et al. Systematic localization of common disease-associated variation in regulatory DNA. Science. 2012;337 10.1126/science.1222794 PMC3771521 22955828

[7] Turner BM (2007). Defining an epigenetic code. Nat Cell Biol.

[8] Heintzman ND, Stuart RK, Hon G, Fu Y, Ching CW, Hawkins RD (2007). Distinct and predictive chromatin signatures of transcriptional promoters and enhancers in the human genome. Nat. Genet. Nature Publishing Group.

[9] Guenther MG, Levine SS, Boyer LA, Jaenisch R, Young RA (2007). A chromatin landmark and transcription initiation at most promoters in human cells. Cell.

[10] Birney E, Stamatoyannopoulos JA, Dutta A, Guigó R, Gingeras TR, Margulies EH (2007). Identification and analysis of functional elements in 1% of the human genome by the ENCODE pilot project. Nature. Nat Publ Group.

[11] Ernst J, Kellis M (2010). Discovery and characterization of chromatin states for systematic annotation of the human genome. Nat Biotechnol Nature Research.

[12] Ernst J, Kheradpour P, Mikkelsen TS, Shoresh N, Ward LD, Epstein CB (2011). Mapping and analysis of chromatin state dynamics in nine human cell types. Nature Nature Research.

[13] Kundaje A, Meuleman W, Ernst J, Bilenky M, Yen A, Heravi-Moussavi A (2015). Integrative analysis of 111 reference human epigenomes. Nature. Nature Research.

[14] Voigt P, Tee W-W, Reinberg D. A double take on bivalent promoters. Genes Dev. 2013;27(12):1318–38. 10.1101/gad.219626.113 10.1101/gad.219626.113 PMC3701188 23788621

[15] Mikkelsen T, Ku M, Jaffe D, Issac B, Lieberman E, Giannoukos G, et al. Genome-wide maps of chromatin state in pluripotent and lineage-committed cells. Nature. Broad Institute of Harvard and MIT, Cambridge, Massachusetts 02142, USA.; 2007;448:553–560. 10.1038/nature06008 PMC2921165 17603471

[16] Mohn F, Weber M, Rebhan M, Roloff T, Richter J, Stadler M, et al. Lineage-specific polycomb targets and de novo DNA methylation define restriction and potential of neuronal progenitors. Mol. Cell. Friedrich Miescher Institute for Biomedical Research, Maulbeerstrasse 66, 4058 Basel, Switzerland.; 2008;30:755–766. 10.1016/j.molcel.2008.05.007 18514006

[17] Roh T-Y, Cuddapah S, Cui K, Zhao K (2006). The genomic landscape of histone modifications in human T cells. Proc Natl Acad Sci U S A.

[18] Barski A, Cuddapah S, Cui K, Roh T-YY, Schones DE, Wang Z (2007). High-resolution profiling of histone methylations in the human genome. Cell.

[19] Cui K, Zang C, Roh T-Y, Schones D, Childs R, Peng W, et al. Chromatin signatures in multipotent human hematopoietic stem cells indicate the fate of bivalent genes during differentiation. Cell Stem Cell. Laboratory of Molecular Immunology, National Heart, Lung, and Blood Institute, National Institutes of Health, 9000 Rockville Pike, Bethesda, MD 20892, USA.; 2009;4:80–93. 10.1016/j.stem.2008.11.011 PMC2785912 19128795

[20] Adli M, Zhu J, Bernstein BE (2010). Genome-wide chromatin maps derived from limited numbers of hematopoietic progenitors. Nat. Methods. Nature Research.

[21] Williams K, Christensen J, Helin K, Blackledge N, Klose R, Bogdanovic O (2011). DNA methylation: TET proteins—guardians of CpG islands?. EMBO Rep EMBO Press.

[22] Voigt P, Tee W-W, Reinberg D. A double take on bivalent promoters. Genes Dev. Howard Hughes Medical Institute, Department of Biochemistry and Molecular Pharmacology, New York University School of Medicine, New York, NY 10016, USA.; 2013;27:1318–1338. 10.1101/gad.219626.113 PMC3701188 23788621

[23] Brookes E, de Santiago I, Hebenstreit D, Morris KJ, Carroll T, Xie SQ (2012). Polycomb associates genome-wide with a specific RNA polymerase II variant, and regulates metabolic genes in ESCs. Cell Stem Cell.

[24] Brookes E, Pombo A (2009). Modifications of RNA polymerase II are pivotal in regulating gene expression states. EMBO Rep.

[25] Gaertner B, Zeitlinger J. RNA polymerase II pausing during development. Development. 2014;141(6):1179–83. 10.1242/dev.088492 10.1242/dev.088492 PMC3943177 24595285

[26] Zeitlinger J, Stark A, Kellis M, Hong J-W, Nechaev S, Adelman K (2007). RNA polymerase stalling at developmental control genes in the Drosophila Melanogaster embryo. Nat Genet Nature Publishing Group.

[27] Krumm A, Hickey LB, Groudine M (1995). Promoter-proximal pausing of RNA polymerase II defines a general rate-limiting step after transcription initiation. Genes Dev.

[28] Gaertner B, Johnston J, Chen K, Wallaschek N, Paulson A, Garruss AS (2012). Poised RNA polymerase II changes over developmental time and prepares genes for future expression. Cell Rep.

[29] Harrow J, Frankish A, Gonzalez JM, Tapanari E, Diekhans M, Kokocinski F (2012). GENCODE: the reference human genome annotation for the ENCODE project. Genome Res.

[30] Xu S, Grullon S, Ge K, Peng W (2014). Spatial clustering for identification of ChIP-enriched regions (SICER) to map regions of histone methylation patterns in embryonic stem cells. Methods Mol Biol Clifton NJ.

[31] Hanazawa M, Narushima H, Minaka N (1995). Generating most parsimonious reconstructions on a tree: a generalization of the Farris-Swofford-Maddison method. Discrete Appl Math North-Holland.

[32] Narushima H, Hanazawa M. A more efficient algorithm for MPR problems in phylogeny. Discrete Appl. Math. North-Holland. 1997;

[33] Mantsoki A, Devailly G, Joshi A (2015). CpG island erosion, polycomb occupancy and sequence motif enrichment at bivalent promoters in mammalian embryonic stem cells. Sci Rep.

[34] Bernstein B, Mikkelsen T, Xie X, Kamal M, Huebert D, Cuff J (2006). A bivalent chromatin structure marks key developmental genes in embryonic stem cells. Cell.

[35] Sánchez-Castillo M, Ruau D, Wilkinson AC, Ng FSL, Hannah R, Diamanti E (2015). CODEX: a next-generation sequencing experiment database for the haematopoietic and embryonic stem cell communities. Nucleic Acids Res.

[36] Anguita E, Hughes J, Heyworth C, Blobel GA, Wood WG, Higgs DR (2004). Globin gene activation during haemopoiesis is driven by protein complexes nucleated by GATA-1 and GATA-2. EMBO J.

[37] Soler E, Andrieu-Soler C, de Boer E, Bryne JC, Thongjuea S, Stadhouders R (2010). The genome-wide dynamics of the binding of Ldb1 complexes during erythroid differentiation. Genes Dev Cold Spring Harbor Laboratory Press.

[38] Vicente C, Conchillo A, García-Sánchez MA, Odero MD (2012). The role of the GATA2 transcription factor in normal and malignant hematopoiesis. Crit Rev Oncol Hematol.

[39] Heinz S, Benner C, Spann N, Bertolino E, Lin YC, Laslo P (2010). Simple combinations of lineage-determining transcription factors prime cis-regulatory elements required for macrophage and B cell identities. Mol Cell.

[40] Najand N, Ryu J-R, Brook WJ. In Vitro site selection of a consensus binding site for the Drosophila Melanogaster Tbx20 homolog midline. Foulkes NS, editor. PLoS One 2012;7:e48176. 10.1371/journal.pone.0048176 PMC3485041 23133562

[41] Ding Y, Lorenz WA, Chuang JH (2012). CodingMotif: exact determination of overrepresented nucleotide motifs in coding sequences. BMC Bioinformatics.

[42] Zhao XD, Han X, Chew JL, Liu J, Chiu KP, Choo A (2007). Whole-genome mapping of histone H3 Lys4 and 27 trimethylations reveals distinct genomic compartments in human embryonic stem cells. Cell Stem Cell.

[43] Muse GW, Gilchrist DA, Nechaev S, Shah R, Parker JS, Grissom SF (2007). RNA polymerase is poised for activation across the genome. Nat. Genet. Nature Publishing Group.

[44] Williams LH, Fromm G, Gokey NG, Henriques T, Muse GW, Burkholder A (2015). Pausing of RNA polymerase II regulates mammalian developmental potential through control of signaling networks. Mol Cell.

[45] Marks H, Kalkan T, Menafra R, Denissov S, Jones K, Hofemeister H (2012). The transcriptional and epigenomic foundations of ground state pluripotency. Cell.

[46] Min IM, Waterfall JJ, Core LJ, Munroe RJ, Schimenti J, Lis JT (2011). Regulating RNA polymerase pausing and transcription elongation in embryonic stem cells. Genes Dev.

[47] Tee W-W, Shen SS, Oksuz O, Narendra V, Reinberg D (2014). Erk1/2 activity promotes chromatin features and RNAPII phosphorylation at developmental promoters in mouse ESCs. Cell.

[48] Kanehisa M, Sato Y, Kawashima M, Furumichi M, Tanabe M (2016). KEGG as a reference resource for gene and protein annotation. Nucleic Acids Res Oxford University Press.

[49] De Gobbi M, Garrick D, Lynch M, Vernimmen D, Hughes JR, Goardon N (2011). Generation of bivalent chromatin domains during cell fate decisions. Epigenetics Chromatin.

[50] Xu J, Carter AC, Gendrel A-V, Attia M, Loftus J, Greenleaf WJ (2017). Landscape of monoallelic DNA accessibility in mouse embryonic stem cells and neural progenitor cells. Nat Genet.

[51] Core LJ, Waterfall JJ, Lis JT (2008). Nascent RNA sequencing reveals widespread pausing and divergent initiation at human promoters. Science.

[52] Yusuf B, Gopurappilly R, Dadheech N, Gupta S, Bhonde R, Pal R (2013). Embryonic fibroblasts represent a connecting link between mesenchymal and embryonic stem cells. Develop Growth Differ.

[53] Takahashi K, Yamanaka S (2006). Induction of pluripotent stem cells from mouse embryonic and adult fibroblast cultures by defined factors. Cell.

[54] Liu J, Wu X, Zhang H, Pfeifer GP, Lu Q (2017). Dynamics of RNA polymerase II pausing and bivalent histone H3 methylation during neuronal differentiation in brain development. Cell Rep.

[55] Dao P, Wojtowicz D, Nelson S, Levens D, Ups PTM (2016). Downs of poised RNA polymerase II in B-cells. Singh M. PLoS Comput Biol.

[56] Barrett T, Wilhite SE, Ledoux P, Evangelista C, Kim IF, Tomashevsky M (2013). NCBI GEO: archive for functional genomics data sets--update. Nucleic Acids Res.

[57] Langmead B, Salzberg SL (2012). Fast gapped-read alignment with bowtie 2. Nat Methods Nature Research.

[58] Li H, Handsaker B, Wysoker A, Fennell T, Ruan J. The sequence alignment/map format and {SAMtools}. 2009; 10.1093/bioinformatics/btp352 PMC2723002 19505943

[59] Zhang Y, Liu T, Meyer CA, Eeckhoute J, Johnson DS, Bernstein BE (2008). Model-based analysis of ChIP-Seq (MACS). Genome Biol.

[60] Trapnell C, Pachter L, Salzberg SL (2009). TopHat: discovering splice junctions with RNA-Seq. Bioinforma Oxf Engl.

[61] Trapnell C, Williams BA, Pertea G, Mortazavi A, Kwan G, van Baren MJ (2010). Transcript assembly and quantification by RNA-Seq reveals unannotated transcripts and isoform switching during cell differentiation. Nat Biotechnol.

[62] Harrow J, Frankish A, Gonzalez JM, Tapanari E. {GENCODE:} The reference human genome annotation for The {ENCODE} Project. 2012; 10.1101/gr.135350.111 PMC3431492 22955987

[63] Quinlan AR, Hall IM (2010). {BEDTools:} a flexible suite of utilities for comparing genomic features. Bioinformatics. Oxford Univ Press.

[64] Müllner D (2013). Fastcluster : fast hierarchical, agglomerative clustering routines for *R* and *Python*. J Stat Softw.

[65] Kishore K, de Pretis S, Lister R, Morelli MJ, Bianchi V, Amati B (2015). methylPipe and compEpiTools: a suite of R packages for the integrative analysis of epigenomics data. BMC Bioinformatics.

[66] Huber W, Carey VJ, Gentleman R, Anders S, Carlson M, Carvalho BS (2015). Orchestrating high-throughput genomic analysis with Bioconductor. Nat Methods Nature Research.

[67] Team RDC (2016). R: a language and environment for statistical computing.

[68] Leisch F, Friedrich. A toolbox for -centroids cluster analysis. Comput. Stat. Data Anal. Elsevier Science Publishers B. V.; 2006;51:526–544.

[69] Gregory R. Warnes, Ben Bolker, Lodewijk Bonebakker, Robert Gentleman, Wolfgang Huber Andy Liaw, Thomas Lumley, Martin Maechler, Arni Magnusson, Steffen Moeller, Marc Schwartz BV. CRAN - Package gplots. 2016.

[70] Alexa A, Rahnenführer J (2016). Gene set enrichment analysis with topGO.

[71] Paradis E, Claude J, Strimmer K (2004). APE: analyses of Phylogenetics and evolution in R language. Bioinforma Oxf Engl Oxford University Press.

[72] Saitou N, Nei M (1987). The neighbor-joining method: a new method for reconstructing phylogenetic trees. Mol. Biol. Evol.

[73] Studier JA, Keppler KJ (1988). A note on the neighbor-joining algorithm of Saitou and Nei. Mol Biol Evol.

[74] Gu Z, Gu L, Eils R, Schlesner M, Brors B (2014). Circlize implements and enhances circular visualization in R. Bioinforma. Oxf. Engl..

[75] Lawrence M, Huber W, Pagès H, Aboyoun P, Carlson M, Gentleman R, et al. Software for computing and annotating genomic ranges. Prlic A, editor. PLoS Comput. Biol. Public Libr Sci; 2013;9:e1003118. 10.1371/journal.pcbi.1003118 PMC3738458 23950696

