# Supplementary material for: Dynamics of promoter bivalency and RNAP II pausing in mouse stem and differentiated cells
Source: BMC Dev Biol. 2018 Feb 20;18:2. doi: 10.1186/s12861-018-0163-7 (PMC5819258; doi:10.1186/s12861-018-0163-7)
Supplement: Supplementary file 1 — Supplementary tables and figures for Mantsoki et al. 2018. (DOCX 1190 kb) [file 12861_2018_163_MOESM1_ESM.docx]

**Supplementary figures and tables for**

**Dynamics of promoter bivalency and RNAP II pausing in mouse stem and differentiated cells**

**Anna Mantsoki, Guillaume Devailly, Anagha Joshi**


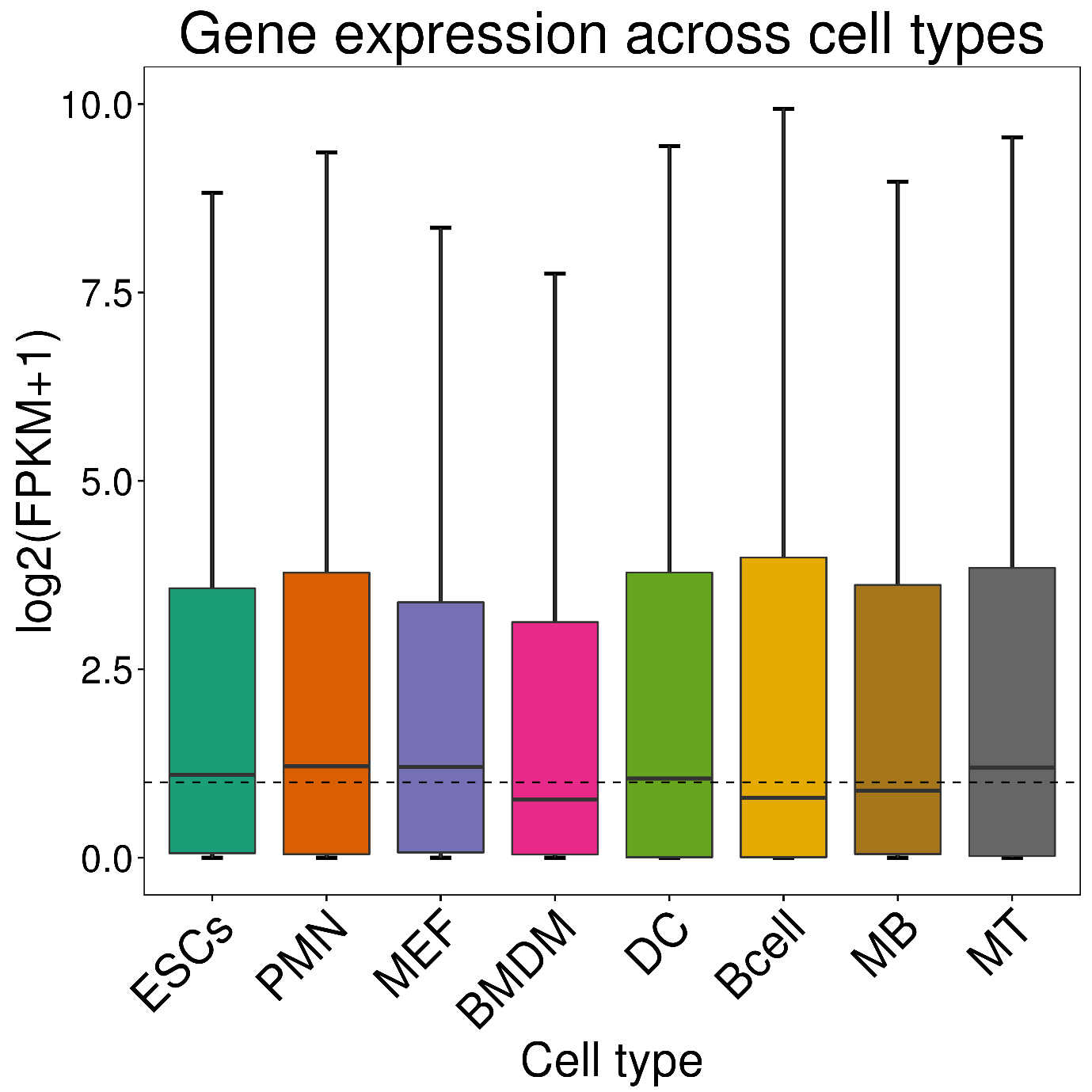


**Figure S1.** Gene expression distribution for all the genes used in the study (22,179) across cell types. The dashed line at **log2(FPKM+1) = 1** represents the threshold imposed to classify genes as expressed and not-expressed.


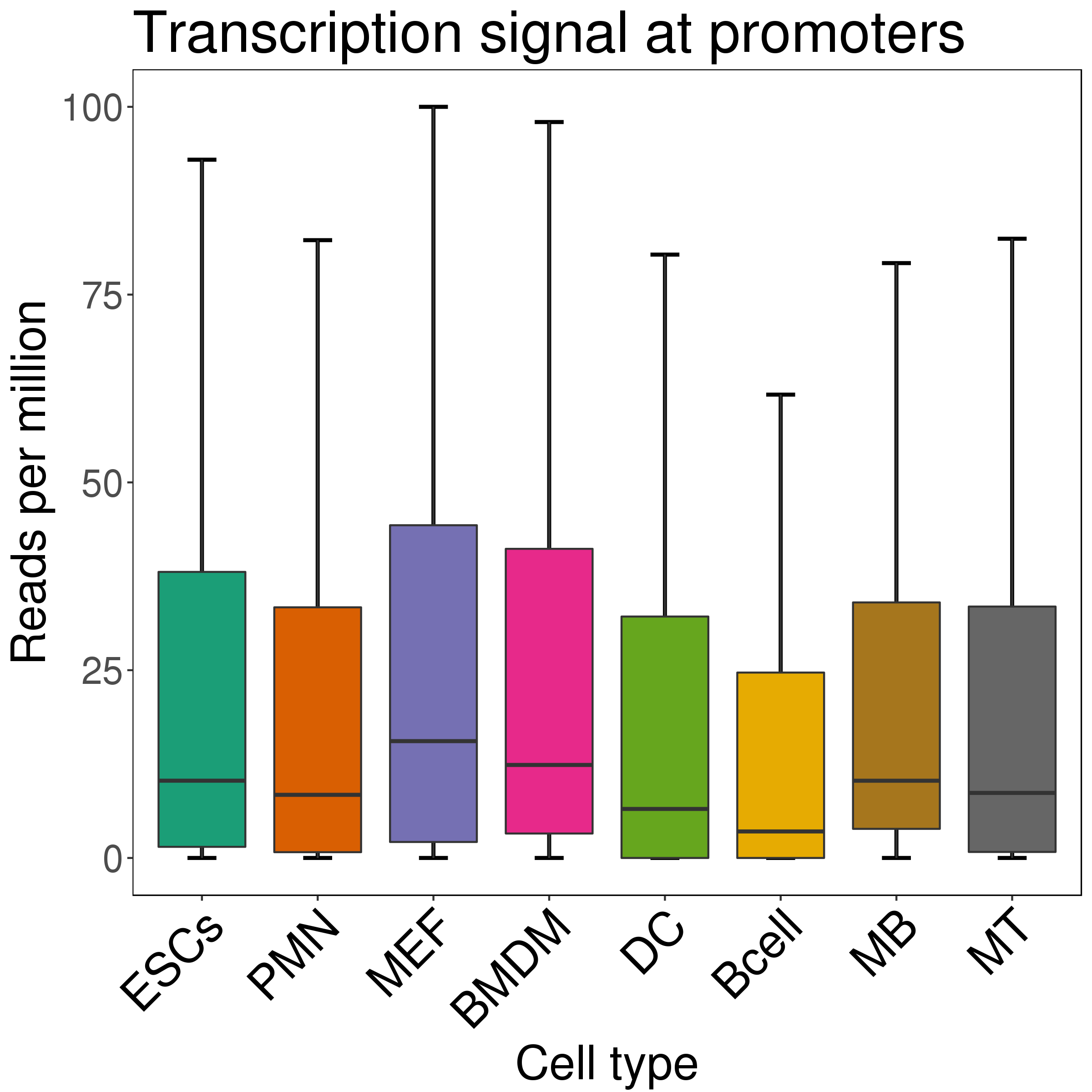


**Figure S2** Transcriptional signal (from RNA-seq datasets) at the promoter areas (-5KB,+5KB) for all the genes used in the study (22,179) across cell types.


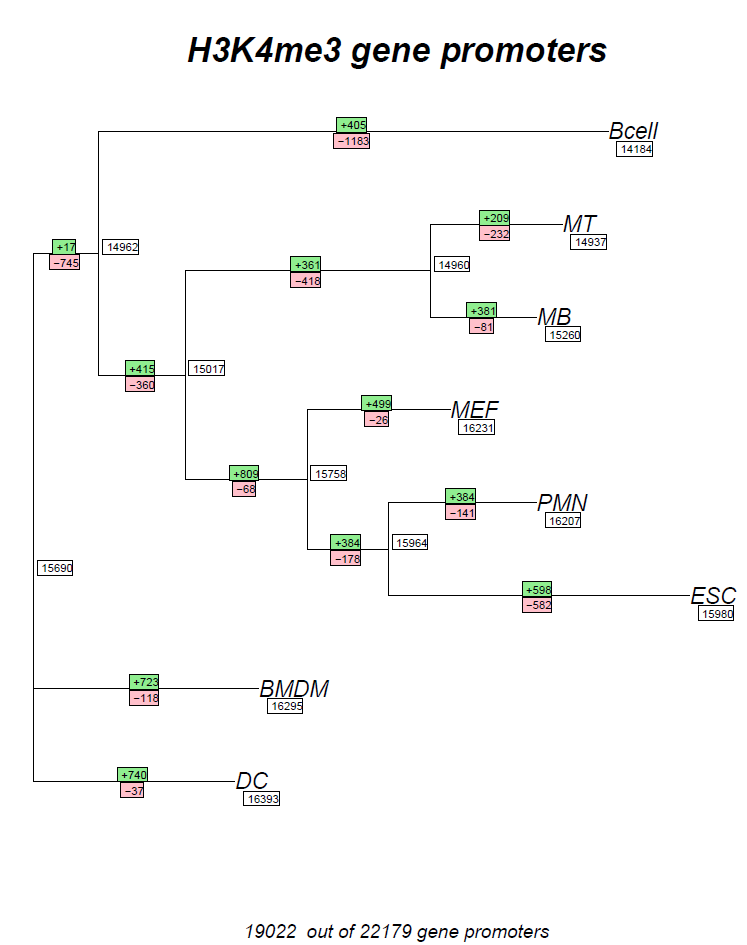


**Figure S3.** H3K4me3 dynamics at the promoter regions (-5Kb, +5Kb) across the cell types. More than 80% promoters (15,690 shared out of 19,022 promoters with H3K4me3 mark across all 8 cell types) retain H3K4me3 modification across cell types.


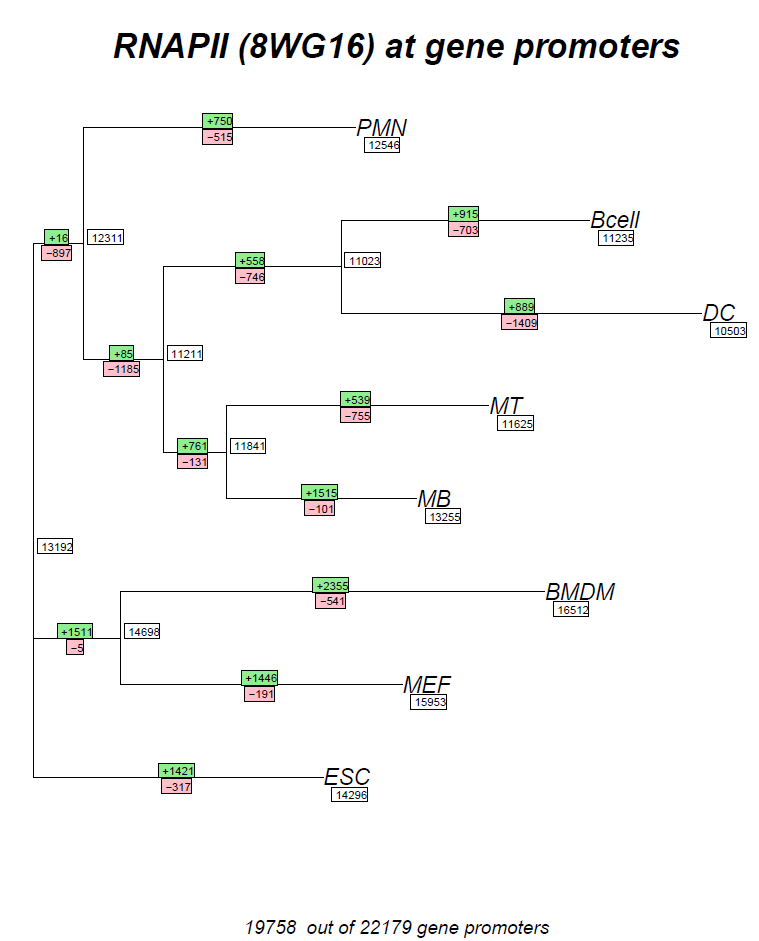


**Figure S4.** RNAPII (8WG16) dynamics at the promoter regions (-5Kb, +5Kb) across the cell types. More than 66% promoters (13,192 shared out of 19,758 promoters with RNAPII binding across all 8 cell types) retain RNAPII binding across cell types.


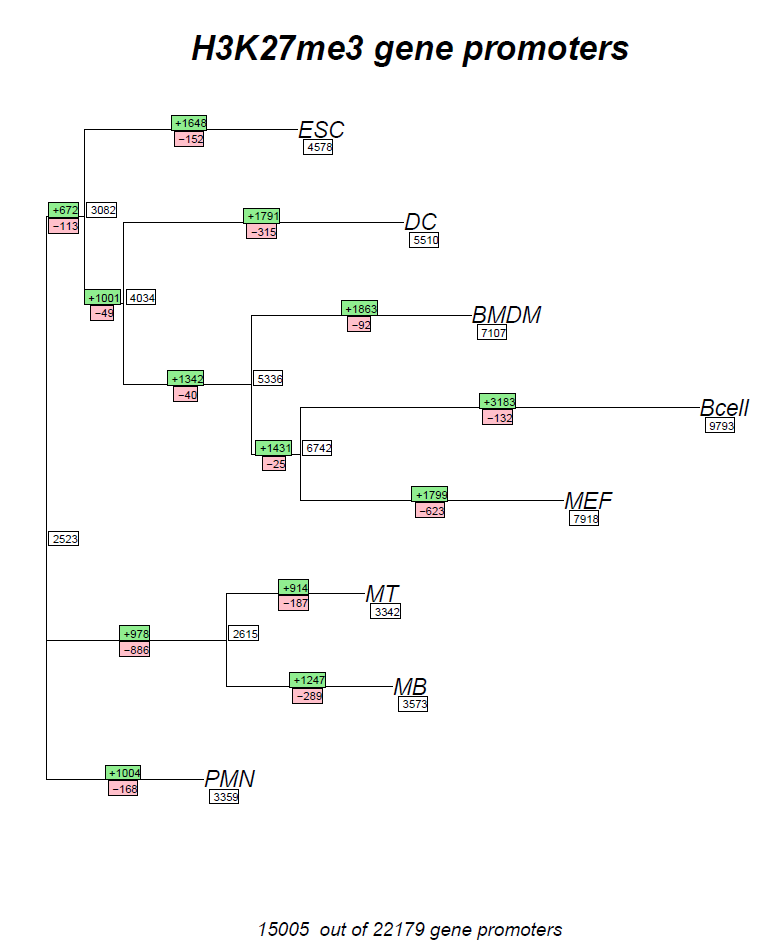


**Figure S5.** H3K27me3 dynamics at the promoter regions (-5Kb, +5Kb) across the cell types. Only about 17% promoters (2,523 shared out of 3,359 promoters with H3K27me3 mark across all 8 cell types) retain H3K27me3 modification across cell types.


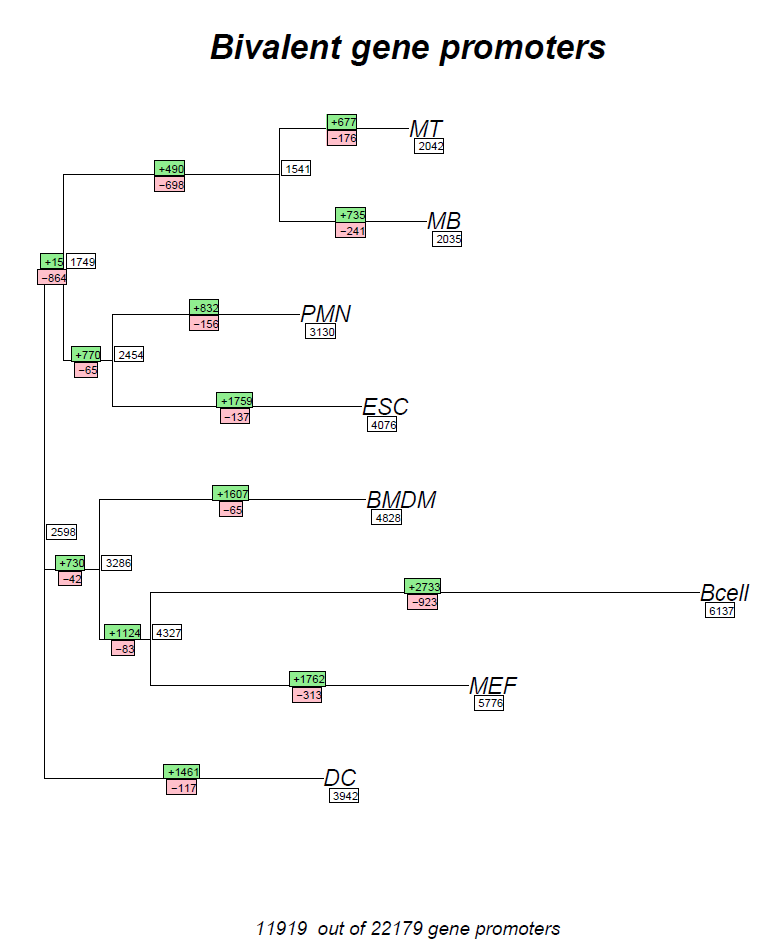


**Figure S6.** Bivalency dynamics at the promoter regions (-5Kb, +5Kb) across the cell types. Only about 22% promoters (2,598 shared out of 11,919 promoters with H3K27me3 mark across all 8 cell types) retain H3K27me3 modification across cell types.

**Figure S7. A)** Boxplot of normalised CpG density across promoters for each of the 9 major profile sub-groups. The groups are ordered from the highest to the lowest according to the mean CpG density across the clusters belonging in that sub-group (the line at the middle of the boxplots denotes the median of the distribution). **B**) Boxplot of normalised CpG density across promoters for each of the 31 clusters. The clusters are ordered from the highest to the lowest according to the mean CpG density across the promoters belonging in that cluster (the line at the middle of the boxplots denotes the median of the distribution. **C**) Percent of overlap of the promoter regions (± 500 bp from the TSS) in each cluster with CpG islands in the mouse genome.


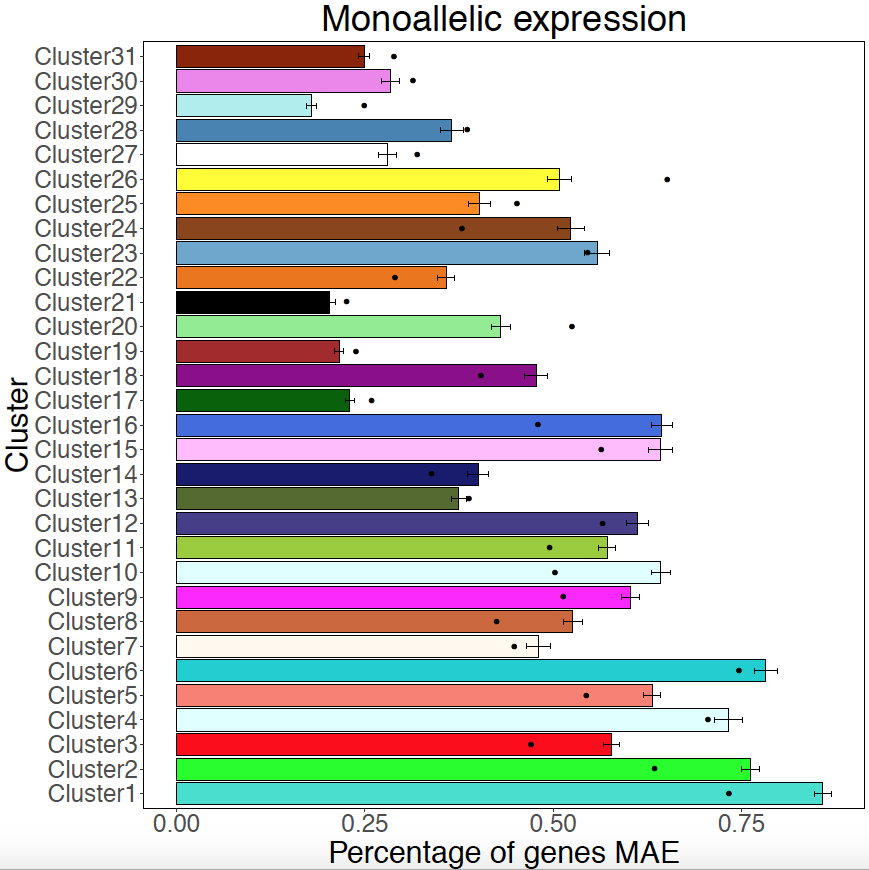


**Figure S8.** Percentage of genes in each cluster overlapping with lists of monoallelically expressed (MAE) genes. The bar shows overlap with predicted MAE genes from dbMAE (Savova et. al, 2016) and the black dot declares the percentage of overlap with experimentally confirmed MAE genes in Lymphoblasts.


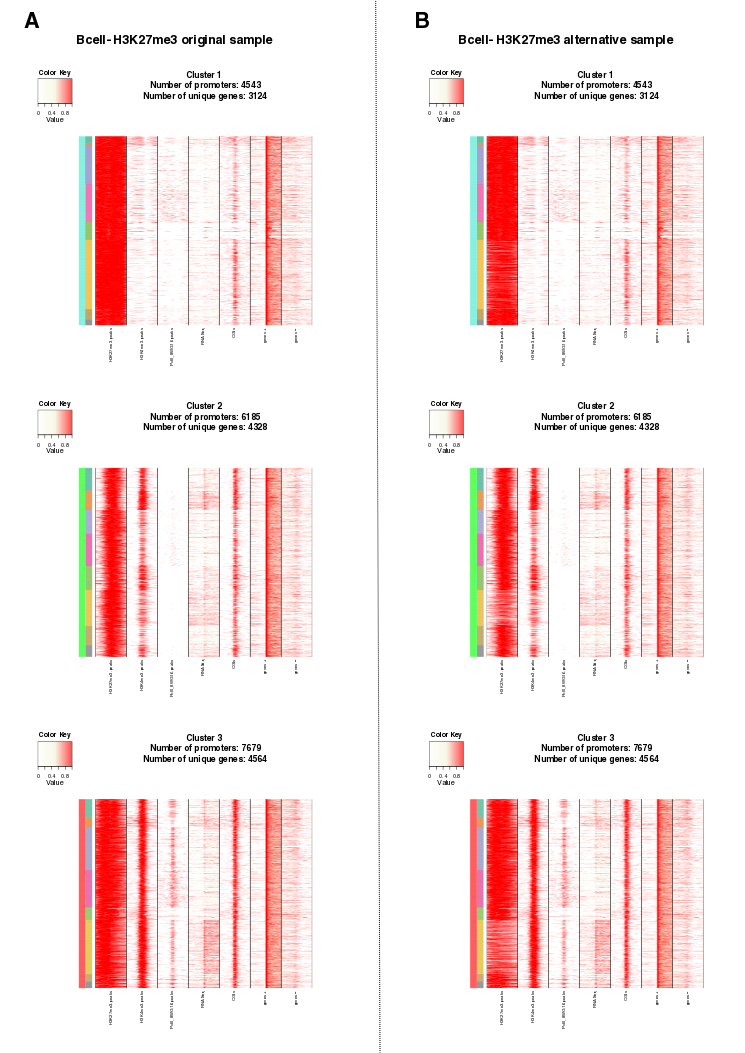


**Figure S9. A**) Representative heatmaps for clusters 1, 2 and 3, which show extremely high levels of H3K27me3 across the 10 kb regions flanking the TSS of the genes. These profiles are plotted using the H3K27me3 sample from B cells that was originally used throughout the study. **B**) We replaced the H3K27me3 sample in B cells with another replicate to assess if there are extremes differences at the signal. There is a minimal decrease, but not significant enough since the H3K27me3 is still strong.

**Supplementary Tables**

**Tables:**

**Table S1**. ChIP-seq samples, accession numbers, cell types, and antibodies used

**Table S2**. The total number of reads, reads mapped to the genome and number of peaks called for each sample.

**Table S3**. RNA-seq samples, accession numbers and cell types

**Table S4.** Classification of 22,179 gene promoters in each cell type according to 1) expression levels (Expressed when log2 (FPKM+1) >1), 2) H3K4me3 marks, 3) H3K4me3 only – H3K4me3 marks that do not overlap with H3K27me3 marks, 4) H3K27me3 marks, 5) H3K27me3 only – H3K27me3 marks that do not overlap with H3K4me3 marks, 6) Bivalent marks – H3K4me3 and H3K27me3 peaks overlapping at the region, 7) RNAPII bound promoters

| Signature | ESCs | PMN | MEF | BMDM | DC | B cell | MB | MT |
| --- | --- | --- | --- | --- | --- | --- | --- | --- |
| Expressed | 11372 | 11604 | 11575 | 10582 | 11227 | 10723 | 10894 | 11451 |
| Not  expressed | 10807 | 10575 | 10604 | 11597 | 10952 | 11456 | 11285 | 10728 |
| H3K4me3  marked | 15980 | 16207 | 16231 | 16295 | 16393 | 14184 | 15260 | 14937 |
| Not H3K4me3  marked | 6199 | 5972 | 5948 | 5884 | 5786 | 7995 | 6919 | 7242 |
| H3K4me3 only  marked | 11904 | 13077 | 10455 | 11467 | 12451 | 8047 | 13225 | 12895 |
| Not H3K4me3  only marked | 10275 | 9102 | 11724 | 10712 | 9728 | 14132 | 8954 | 9284 |
| H3K27me3  marked | 4578 | 3359 | 7918 | 7107 | 5510 | 9793 | 3573 | 3342 |
| Not H3K27me3  marked | 17601 | 18820 | 14261 | 15072 | 16669 | 12386 | 18606 | 18837 |
| H3K27me3 only  marked | 502 | 229 | 2142 | 2279 | 1568 | 3656 | 1538 | 1300 |
| Not H3K27me3  only marked | 21677 | 21950 | 20037 | 19900 | 20611 | 18523 | 20641 | 20879 |
| Bivalent | 4076 | 3130 | 5776 | 4828 | 3942 | 6137 | 2035 | 2042 |
| Not  bivalent | 18103 | 19049 | 16403 | 17351 | 18237 | 16042 | 20144 | 20137 |
| RNAPII  bound | 14296 | 12546 | 15953 | 16512 | 10503 | 11235 | 13255 | 11625 |
| Not RNAPII  bound | 7883 | 9633 | 6226 | 5667 | 11676 | 10944 | 8924 | 10554 |

**Table S5.** 31 clusters ordered by how they are displayed in the heatmap in Figure 2A. Total number of gene promoters-cell type in each cluster, unique number of gene promoters in each cluster independently of cell type information, and ratio of unique to total number of genes. The clusters in black background displayed a ratio lower than 0.6, indicating clusters with gene promoter signatures conserved across cell types.

| **Cluster** | **Total number of genes** | **Unique genes** | **Ratio**  **Unique/total number of gene promoters** |
| --- | --- | --- | --- |
| 1 | 4543 | 3124 | 0.6876513 |
| 2 | 6185 | 4328 | 0.6997575 |
| **3** | **7679** | **4564** | **0.5943482** |
| 4 | 1400 | 1140 | 0.8142857 |
| 5 | 3275 | 2649 | 0.808855 |
| 6 | 823 | 679 | 0.8250304 |
| 7 | 158 | 118 | 0.7468354 |
| 8 | 105 | 100 | 0.952381 |
| 9 | 1464 | 1236 | 0.8442623 |
| 10 | 486 | 393 | 0.808642 |
| 11 | 137 | 134 | 0.9781022 |
| 12 | 289 | 254 | 0.8788927 |
| 13 | 924 | 615 | 0.6655844 |
| 14 | 196 | 166 | 0.8469388 |
| 15 | 687 | 514 | 0.7481805 |
| 16 | 304 | 262 | 0.8618421 |
| 17 | 3002 | 2396 | 0.7981346 |
| 18 | 212 | 161 | 0.759434 |
| **19** | **54580** | **13185** | **0.241572** |
| **20** | **1520** | **663** | **0.4361842** |
| **21** | **12250** | **5854** | **0.4778776** |
| 22 | 361 | 309 | 0.8559557 |
| 23 | 166 | 109 | 0.6566265 |
| **24** | **285** | **149** | **0.522807** |
| 25 | 115 | 72 | 0.626087 |
| **26** | **9746** | **3750** | **0.3847732** |
| 27 | 124 | 103 | 0.8306452 |
| 28 | 118 | 97 | 0.8220339 |
| 29 | 3428 | 2212 | 0.6452742 |
| 30 | 1705 | 1047 | 0.6140762 |
| 31 | 474 | 406 | 0.8565401 |

**Table S6.** Unique TFs enriched specifically in Cluster 3 or Cluster 5 (P value < 0.05)

| Cluster | Unique TFs enriched |
| --- | --- |
|  |  |
| Cluster3 | Rbbp5, Pou5f1, Sin3A, Nanog, Tcf3, Prep, Chd4, HoxB4, Cbx8, Runx1, Rbpj, Gfi1b, PU.1, Mxi1, Gfi1b, Sfpi1, Cebpb, Runx2, Jun, p300 |
| Cluster5 | Ebf1, Ldb1, Stat5B, E2f1, Irf1, Maff, Rel, Egr1, Rad21, Stat3, Ncoa3, Tfe3, Sox2, Tal1, Ctcf, Cebpa, Rela, Meis1, Scl, Gata6, Eto2, Ldb1, Mtgr1, Ascl2, Fosl2 |
